# CircPVT1 Promotes Lung Metastasis and Tumor Progression in Renal Cell Carcinoma by Encoding the cP104aa Peptide and Targeting EIF4A3

**DOI:** 10.1002/advs.202501211

**Published:** 2025-09-18

**Authors:** Houliang Zhang, Tao Tao, Jie Ji, Tonglei Zhao, Si Sun, Lijie Zhang, Jianping Wu, Ming Chen, Shuqiu Chen, Bin Xu, Weipu Mao

**Affiliations:** ^1^ Department of Urology Affiliated Zhongda Hospital of Southeast University Nanjing 210009 China; ^2^ Department of Urology The First Affiliated Hospital of USTC Division of Life Sciences and Medicine University of Science and Technology of China Hefei 230001 China; ^3^ Department of Urology Nanjing Lishui District People's Hospital Zhongda Hospital Lishui Branch Southeast University Nanjing 211200 China; ^4^ Surgical Research Center Institute of Urology Southeast University Medical School Nanjing 210009 China

**Keywords:** axitinib, circPVT1, cP104aa, EIF4A3, renal cell carcinoma

## Abstract

Circular RNA (circRNA) plays a pivotal role in the pathogenesis of renal cell carcinoma (RCC). CircRNAs regulate gene expression via RNA‐binding proteins (RBPs) and also exert biological effects through peptide encoding. CircPVT1 has been previously identified as an oncogenic circRNA. This study identified that circPVT1 encodes a 104‐amino acid peptide, termed cP104aa. Functional assays showed that circPVT1 and the cP104aa peptide enhance RCC cell proliferation, invasion, migration, and lung metastasis both in vitro and in vivo. Mechanistically, the cP104aa peptide interacts with HNRNPK, leading to reduced c‐MYC ubiquitination and increased c‐MYC expression. Additionally, circPVT1 directly associates with EIF4A3, facilitating the expression of the target gene c‐MYC. Furthermore, axitinib is shown to target the degradation of the cP104aa peptide. These findings reveal a novel mechanism by which circPVT1 contributes to RCC, highlighting the potential of the cP104aa peptide as a therapeutic target. Axitinib may serve as an effective therapeutic agent for patients with advanced RCC exhibiting high cP104aa expression.

## Graphical abstract

This study reveals that circPVT1 promotes the renal cell carcinoma lung metastasis and tumor progression through encoding the cP104aa peptide and directly binds with EIF4A3, which jointly regulates the target gene c‐MYC.

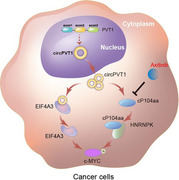

## 1 Introduction

Renal cell carcinoma (RCC) is the most common form of kidney cancer, accounting for 90% of cases.^[^ ^1^ ^]^ Surgical intervention remains the primary treatment option for RCC due to resistance to radiotherapy and chemotherapy.^[^ ^2^ ^]^ However, 30% of patients experience recurrence or metastasis post‐surgery, leading to poor prognosis.^[^ ^3^ ^]^ The underlying pathogenesis of RCC remains unclear, and reliable tumor biomarkers have yet to be identified. Consequently, identifying potential biomarkers and elucidating new mechanisms involved in RCC progression and metastasis is critical, with the development of targeted therapies becoming a high priority for treatment.

Circular RNAs (circRNAs), a novel class of non‐coding RNAs (ncRNAs), have attracted significant attention in recent years.^[^ ^4^ ^]^ Due to their high expression, tissue or cell specificity, and remarkable stability, circRNAs are emerging as promising biomarkers and therapeutic targets.^[^ ^5^ ^]^ Growing evidence suggests that circRNAs are not merely by‐products of splicing errors but actively participate in various physiological and pathological processes.^[^ ^6^ ^]^ They exert biological functions through mechanisms such as acting as miRNA sponges^[^ ^7^ ^]^ or binding to proteins.^[^ ^8^ ^]^ Certain circRNAs, containing internal ribosome entry sites (IRESs) and open reading frames (ORFs), have the potential to encode peptides or proteins.^[^ ^9^ ^]^ For example, circFNDC3B inhibits colon cancer progression by regulating Snail through the encoding of circFNDC3B‐218aa.^[^ ^10^ ^]^ Additionally, other circRNAs, such as circMTHFD2L and circDIDO1, encode peptides or proteins, further expanding our understanding of their roles in cancer.^[^ ^11^ ^]^ However, the involvement of circRNAs in RCC tumorigenesis and development through peptide encoding remains unexplored.

CircPVT1 (hsa_circ_0001821) is a circRNA generated from the cyclization of exon 2 of the PVT1 gene (chr8: 128902834–128903244).^[^ ^12^ ^]^ Its expression has been reported to be upregulated in various cancers.^[^ ^13^ ^]^ In the present study, circPVT1 levels were significantly elevated in RCC tumors and correlated with poor prognosis. Further in vitro and in vivo experiments demonstrated that circPVT1 significantly enhanced the proliferation, migration, and invasion of RCC cells. Mechanistically, circPVT1 promotes RCC progression through two distinct mechanisms: by encoding a novel 104‐amino acid peptide, cP104aa, via IRES‐dependent translation, which subsequently upregulates c‐MYC expression by binding to heterogeneous nuclear ribonucleoprotein K (HNRNPK), and by directly binding to EIF4A3 to increase c‐MYC expression. Additionally, axitinib was identified as a potential therapeutic agent capable of reversing RCC progression by targeting the degradation of the cP104aa peptide, offering a potential approach for personalized therapy. Thus, our findings suggest that circPVT1 and the cP104aa peptide can serve as biomarkers for assessing tumor progression and as potential targets for advanced RCC treatment.

## 2 Results

### 2.1 CircPVT1 is Highly Expressed in RCC Tumors and is Associated with Poor Prognosis

**Figure 1:**
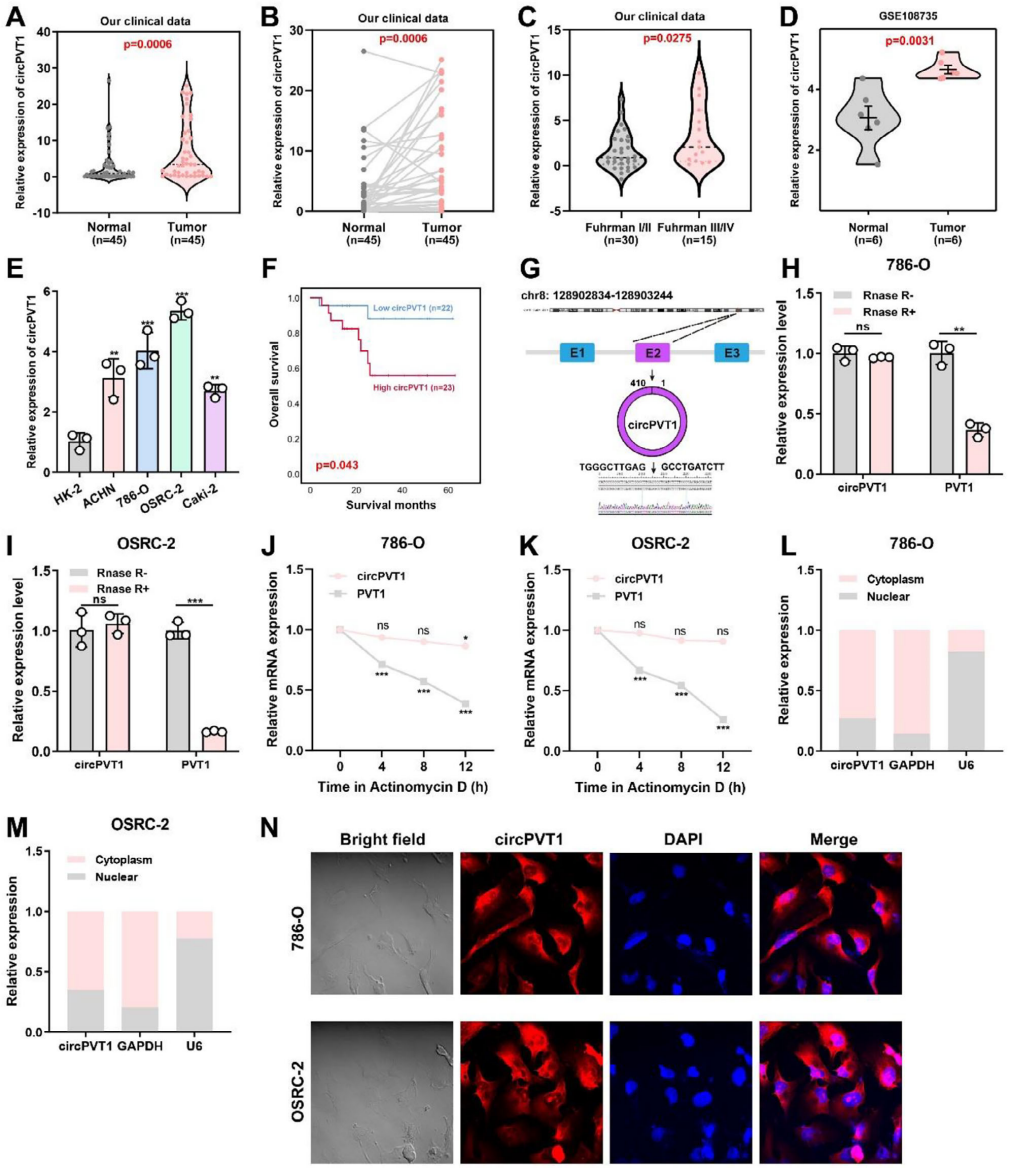
Characterization of circPVT1 in RCC tissues and cell lines. A, B) CircPVT1 exhibited higher expression in tumor tissues (*n* = 45) than in adjacent normal tissues (*n* = 45). C) Expression analysis of circPVT1 in different Fuhrman grades in our patient cohort (*n* = 45). D) Relative expression of circPVT1 in tumor tissues (*n* = 6) and adjacent normal tissues (*n* = 6) from GSE108735. E) Expression levels of circPVT1 in RCC cell lines quantified through qRT‐PCR. F) Kaplan–Meier survival curves showed the correlations between circPVT1 expression and OS. Expression of circPVT1 in RCC cell lines was quantified using qRT‐PCR. G) Schematic illustration of circPVT1 produced from exon 2 of *PVT1*. H, I) qRT‐PCR analysis of circPVT1 and linear PVT1 in RCC cells treated with RNase R. J, K) mRNA stability of circPVT1 and PVT1 in 786‐O and OSRC‐2 cells following actinomycin D treatment was determined through qRT‐PCR. L, M) Expression levels of cytoplasmic control transcripts (GAPDH), the nuclear control transcript (U6), and circPVT1 were determined through qRT‐PCR in cytoplasmic and nuclear fractions of RCC cells. N) FISH was conducted to determine the subcellular localization of circPVT1. Scale bar: 10 µm. N.S.: not significant. Statistical significance is indicated (*p < 0.05, **p < 0.01, ***p < 0.001) by Student's t‐test or ANOVA.

The expression of circPVT1 was examined in 45 RCC tumors and adjacent normal tissues using qRT‐PCR analysis. The results indicated that circPVT1 expression was significantly higher in RCC tumor tissues than in adjacent normal tissues (**Figure** **1**A,B). Furthermore, a significant positive correlation was observed between circPVT1 expression and the Fuhrman grade in our clinical data (Figure 1C). The upregulation of circPVT1 in RCC was also confirmed using the GSE108735 dataset (Figure 1D). Additionally, circPVT1 expression was elevated in RCC cell lines (ACHN, 786‐O, OSRC‐2, and Caki‐2) compared to the normal urinary epithelial cell line HK‐2 (Figure 1E). Kaplan‐Meier survival analysis revealed that high circPVT1 expression was associated with poor prognosis in patients with RCC (Figure 1F). CircPVT1, originating from exon 2 of the *PVT1* gene, has a full length of 401 bp. The back‐splicing junction of circPVT1 was confirmed through Sanger sequencing (Figure 1G). In comparison to the linear *PVT1* mRNA, circPVT1 exhibited resistance to RNase R degradation (Figure 1H,I) and a longer half‐life (Figure 1J,K) in 786‐O and OSRC‐2 cells. Further, qRT‐PCR analysis of cell fractions indicated that circPVT1 was predominantly localized in the cytoplasm (Figure 1L,M), a result verified by fluorescence in situ hybridization (FISH) (Figure 1N). These findings, derived from both public datasets and our clinical samples, highlight the elevated expression of circPVT1 in RCC and its association with poor prognosis.

### 2.2 CircPVT1 Promotes the Proliferation and Migration of RCC Cells Both In Vitro and In Vivo

**Figure 2:**
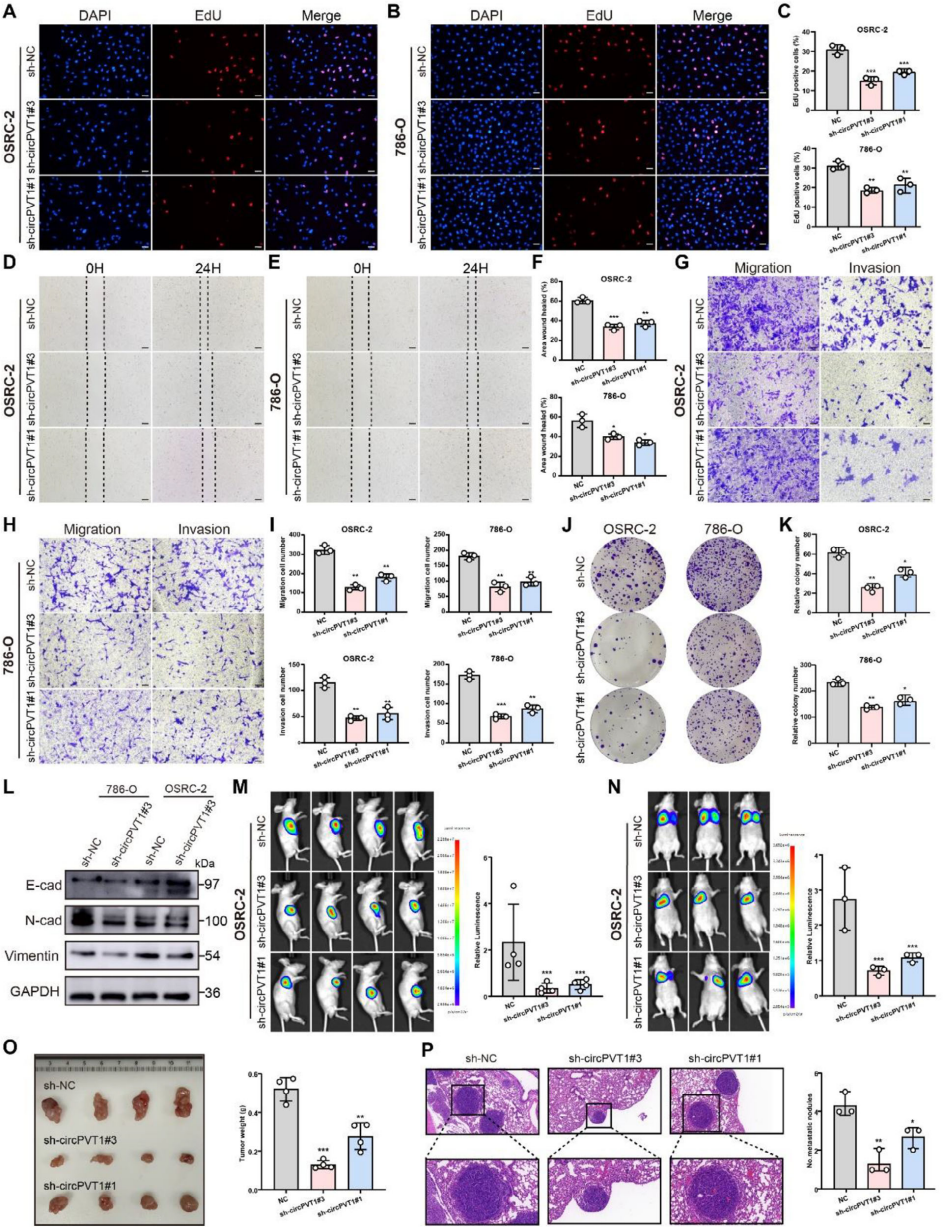
CircPVT1 promotes RCC growth and metastasis in vitro and in vivo. A–C) EdU assay was performed to assess the proliferation of OSRC‐2 and 786‐O cells treated with sh‐circPVT1#3, sh‐circPVT1#1, or sh‐NC. D–F) Wound healing assays were performed in OSRC‐2 and 786‐O cells treated with sh‐circPVT1#3, sh‐circPVT1#1, or sh‐NC. G–I) Transwell migration and invasion assays were performed in OSRC‐2 and 786‐O cells treated with sh‐circPVT1#3, sh‐circPVT1#1, or sh‐NC. J, K) Effect of circPVT1 on proliferation in RCC cell lines was determined through colony formation assay. L) Western blotting of vimentin, N‐cadherin, and E‐cadherin in OSRC‐2 and 786‐O cells treated with circPVT1 knockdown or negative control. M) IVIS imaging of subcutaneous xenograft tumors. N) Representative images of metastasis observed through IVIS. O) Representative images of xenograft tumors in nude mice. P) H&E images of lung tissue sections from the control and sh‐circPVT1 groups.Scale bar: 100 µm. Statistical significance is indicated (**p < 0.01, ***p < 0.001) by Student's t‐test or ANOVA.

To investigate the biological functions of circPVT1, gain‐ and loss‐of‐function assays were performed. CircPVT1 was knocked down using sh‐RNA and overexpressed in 786‐O and OSRC‐2 cells. Stable knockdown of circPVT1 was established based on qRT‐PCR results by selecting the highly efficient sh‐circPVT1#3 and sh‐circPVT1#1, along with overexpression of circPVT1 in 786‐O and OSRC‐2 cells (Figure S1A,B, Supporting Information). EdU assays confirmed that cell proliferation was reduced in the sh‐circPVT1 group compared to the control group (**Figure** **2**A–C). Moreover, wound healing assays demonstrated a slower wound closure rate in the sh‐circPVT1 cells of 786‐O and OSRC‐2 lines than in the control cells (Figure 2D–F). Similarly, knockdown of circPVT1 impaired cell migration and invasion (Figure 2G–I). Colony formation assays further corroborated the role of circPVT1 in promoting cell proliferation (Figure 2J,K). Given that epithelial‐mesenchymal transition (EMT) is a critical process driving RCC invasion and metastasis,^[^ ^14^ ^]^ Western blot (WB) analysis was performed to assess EMT markers. The results showed increased levels of the epithelial marker E‐cadherin, while mesenchymal markers, such as N‐cadherin and vimentin, were reduced in the sh‐circPVT1 group (Figure 2L). These in vitro data collectively suggest that circPVT1 acts as a promoter of RCC progression, regulating cell proliferation, migration, and invasion. To further confirm the in vivo function of circPVT1, OSRC‐2 cells transfected with sh‐circPVT1 were subcutaneously implanted into nude mice. IVIS imaging revealed a significant reduction in xenograft tumor growth upon circPVT1 knockdown (Figure 2M). In line with these findings, silencing circPVT1 resulted in a marked decrease in both tumor size (Figure 2O) and volume (Figure S2A–D, Supporting Information). To assess tumor proliferation and apoptosis, Ki‐67, Bax, and TUNEL staining were performed. Immunohistochemistry (IHC) results showed that circPVT1 knockdown inhibited cell proliferation and promoted apoptosis (FigureS2E, Supporting Information). To investigate the role of circPVT1 in tumor metastasis, luciferase‐labeled OSRC‐2 cells were injected into the tail vein of nude mice. IVIS imaging showed significantly lower luciferase signals in the sh‐circPVT1 group compared to the control group (Figure 2N). Additionally, hematoxylin and eosin (HE) staining confirmed that circPVT1 knockdown suppressed lung metastasis (Figure 2P).

Functional experiments were conducted using circPVT1 overexpression. OE‐circPVT1 cells exhibited higher migratory activity than vector control cells (Figure S3A,B, Supporting Information). Overexpression of circPVT1 further enhanced cell migration (Figure S3C, Supporting Information). Colony formation assays revealed that circPVT1 overexpression significantly promoted cell proliferation (Figure S3D,H, Supporting Information), a result also confirmed by the EdU assay (Figure S3F,G,I, Supporting Information). WB analysis showed that E‐cadherin levels were decreased, while N‐cadherin and vimentin levels were elevated in the OE‐circPVT1 group (Figure S6E, Supporting Information). In vivo experiments confirmed these findings. IVIS imaging indicated that circPVT1 overexpression in OSRC‐2 cells led to increased tumor growth (Figure S4A, Supporting Information). Representative images of xenograft tumors confirmed that circPVT1 acts as a promoter of tumorigenesis in RCC (Figure S4B–E, Supporting Information). IHC results showed that circPVT1 overexpression promoted cell proliferation and inhibited apoptosis (Figure S4F, Supporting Information). To simulate tumor metastasis, OSRC‐2 OE‐circPVT1 cells were injected into the tail vein of nude mice. IVIS imaging revealed increased luciferase signals in the OE‐circPVT1 group, indicating that circPVT1 overexpression facilitated RCC cell metastasis (Figure S5A, Supporting Information). Consistently, HE staining demonstrated an increase in pulmonary nodule metastasis in the OE‐circPVT1 group compared to controls (Figure S5B, Supporting Information).

### 2.3 CircPVT1 Encodes a 104‐Amino Acid Novel Peptide, cP104aa

**Figure 3:**
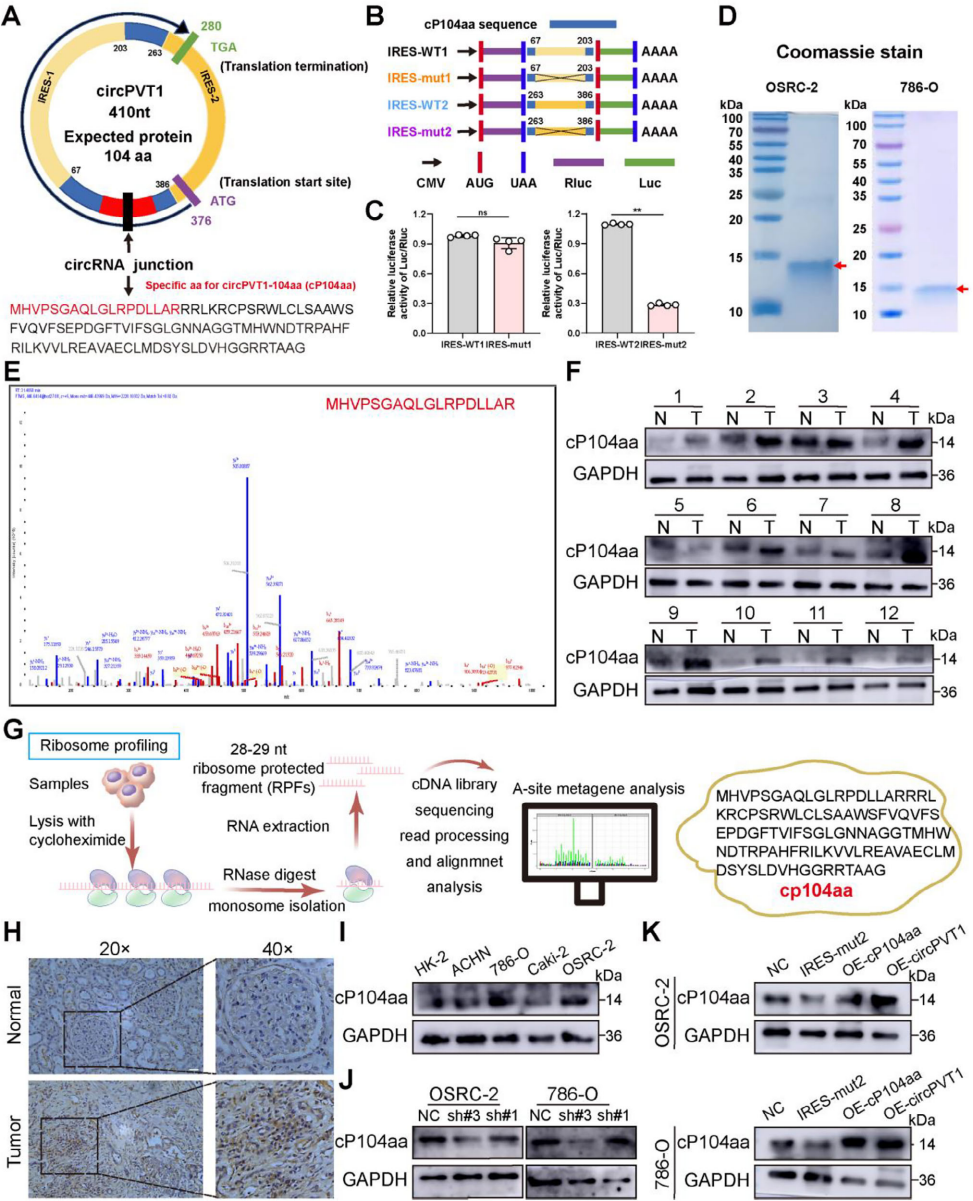
CircPVT1 encodes a novel peptide, cP104aa. A) Upper panel: putative ORF in circPVT1; lower panel: sequences of the putative ORF in circPVT1. B) IRES sequences in circPVT1 or its different truncations cloned between Rluc and Luc reporter genes with independent start and stop codons. C) Relative luciferase activity in the aforementioned vectors. D) Identification of cP104aa in OSRC‐2 and 786‐O cells. An enriched band was identified through IP of cP104aa antibody, electrophoresis, and Coomassie Blue staining at the 14 kDa position. E) Corresponding gel band was excised for LC–MS/MS analysis. F) Expression levels of cP104aa in 12 pairs of RCC and adjacent tissues were determined through WB assays. G) Identifying translated small open reading frames (smORFs) by ribosome profiling (Ribo‐seq). H) Expression of cP104aa in paraffin sections was examined using IHC. I) Expression level of cP104aa in HK‐2 and RCC cell lines. J) Expression level of cP104aa in OSRC‐2 and 786‐O cells transfected with sh‐circPVT1 or sh‐NC. K) Anti‐cP104aa antibodies were used to detect cP104aa in OSRC‐2 and 786‐O cells transfected with control, IRES‐mut2, OE‐cP104aa, and OE‐circPVT1. (t‐tests were used for two groups) Scale bar: 100 µm. N.S.: not significant. Statistical significance is indicated (**p < 0.01) by Student's t‐test or ANOVA.

Emerging evidence suggests that circRNAs are capable of encoding proteins or peptides.^[^ ^15^ ^]^ Analysis of the circRNADb database revealed that circPVT1 contains two IRES sequences and an ORF with an ATG initiation codon, indicating its potential to encode a novel 104‐amino acid peptide, termed cP104aa (**Figure** **3**A). To validate the predicted IRES activity, several Flag‐tagged vectors for circPVT1 were constructed. Luciferase assays demonstrated that the IRES‐mut2 reporter exhibited significantly reduced luciferase activity compared to the wild‐type IRES reporter (Figure 3B,C). Cell lysates from OSRC‐2 and 786‐O cells transfected with Lv‐circPVT1‐Flag were then analyzed, revealing a prominent band at ≈14 kDa by Coomassie staining (Figure 3D). The 14 kDa protein bands were excised and subjected to LC‐MS/MS analysis, identifying the unique sequence of cP104aa (MHVPSGAQLGLRPDLLAR) (Figure 3E). To assess whether cP104aa was expressed in RCC, proteins from tumor and adjacent normal tissues were extracted, and WB analysis confirmed its elevated expression in RCC (Figure 3F). Ribosomal protection fragments (RPFs) were collected and subjected to transcriptional mapping, revealing distinct 3‐nucleotide periodicity in the reads mapped to transcript regions, supporting robust quality control. Ribosome profiling analysis further confirmed that the ORF encoded a 104‐amino acid peptide, designated cP104aa (Figure 3G; Data S1, Supporting Information). To further confirm the coding potential of circPVT1, we constructed the pmirGLO‐cP104aa luciferase reporter that contained cP104aa cDNA. Results of dual‐luciferase assay showed that translation efficiency of cP104aa in OSRC‐2 and 786‐O cells was significantly greater than in sh‐circPVT1#3 OSRC‐2 and 786‐O cells (Figure S6, Supporting Information). IHC performed on tissue samples also showed upregulation of cP104aa in RCC tumors compared to paired adjacent normal tissues (Figure 3H). Additionally, cP104aa expression was significantly higher in various RCC cell lines compared to HK‐2 cells (Figure 3I). Given the potential of circPVT1 to encode cP104aa, further investigation was conducted to determine whether circPVT1 or the encoded peptide cP104aa contributes to tumor promotion in RCC. Expression levels of cP104aa were measured in circPVT1‐knockdown cell lines. WB results revealed that knockdown of circPVT1 significantly reduced cP104aa expression (Figure 3J). Subsequently, stable OSRC‐2 and 786‐O cell lines were generated with the following constructs: IRES‐WT and IRES‐mut2 (overexpressing circPVT1 without cP104aa), Vector#1 and OE‐cP104aa (directly overexpressing cP104aa), Vector#2 and OE‐circPVT1 (overexpressing both circPVT1 and cP104aa). Following transfection, cells in the OE‐cP104aa and OE‐circPVT1 groups showed increased cP104aa expression, whereas cells transfected with IRES‐mut2 displayed decreased expression (Figure 3K).

### 2.4 cP104aa Promotes the Proliferative and Invasive Abilities of RCC In Vitro and In Vivo

**Figure 4:**
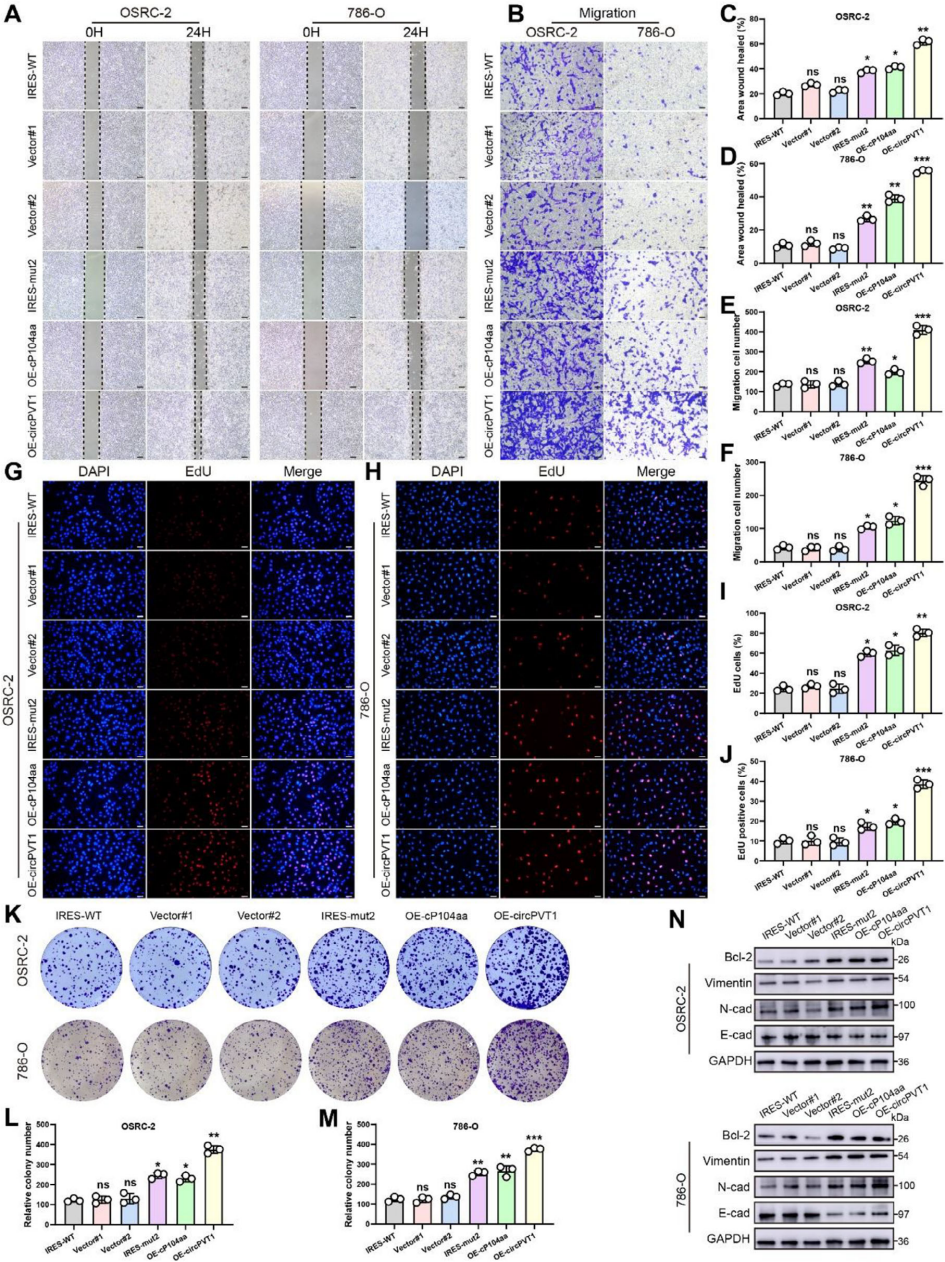
CircPVT1 is a tumor‐promoting factor in RCC cells. A, C, and D) Wound healing assays were conducted in OSRC‐2 and 786‐O cells treated with IRES‐WT, Vector#1, Vector#2, IRES‐mut2, OE‐cP104aa, and OE‐circPVT1. B, E, and F) The Effect of the six aforementioned plasmids on cell migration was examined through a transwell assay. G‐J) EdU assays of OSRC‐2 and 786‐O transfected with six plasmids. K‐M) Colony formation assay was performed to evaluate the proliferation ability of cells transfected with the aforementioned plasmids. N) Expression levels of vimentin, N‐cadherin, E‐cadherin, and Bcl‐2 were determined using WB assays in OSRC‐2 and 786‐O cells. (t‐tests were used for two groups) Scale bar: 100 µm. N.S.: not significant. Statistical significance is indicated (*p < 0.05, **p < 0.01, ***p < 0.001) by Student's t‐test or ANOVA.

Functional experiments were conducted to validate the biological role of cP104aa following transfection with the six aforementioned vectors. Wound healing assays revealed that the IRES‐mut2, cP104aa, and OE‐circPVT1 groups exhibited a higher rate of wound closure compared to the control group (**Figure** **4**A,C,D). Both cP104aa and circPVT1 overexpression enhanced the migratory and invasive capacity of OSRC‐2 and 786‐O cells, as demonstrated by the transwell assay (Figures 4B,E,F; S7, Supporting Information). EdU assays further confirmed that cell proliferation was increased in the IRES‐mut2, OE‐cP104aa, and OE‐circPVT1 transfected groups (Figure 4G–J), with these findings corroborated by colony formation assays (Figure 4K–M). WB analysis showed reduced levels of the epithelial marker E‐cadherin and elevated levels of mesenchymal markers N‐cadherin and vimentin in the cP104aa, IRES‐mut2, and circPVT1 groups (Figure 4N). Collectively, these results confirm that circPVT1 and its encoded cP104aa peptide enhance RCC cell proliferation, migration, and invasion.

**Figure 5:**
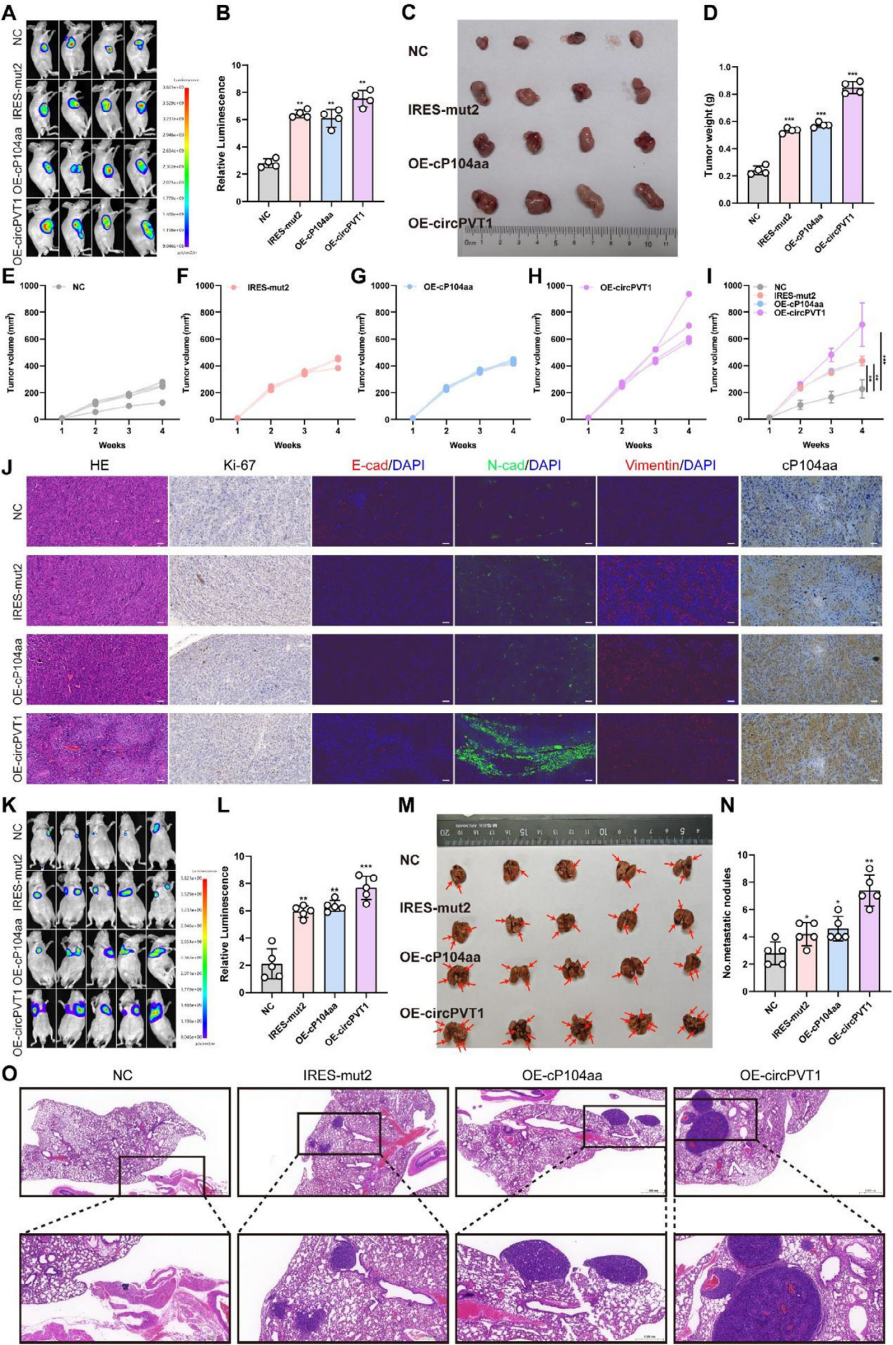
CircPVT1 knockdown inhibits RCC growth and metastasis in vivo. A, B) Bioluminescence imaging of the four groups, with fluorescent quantitative statistics performed on representative images. C‐D) Representative images of xenograft tumors in nude mice and the average tumor weight of nude mice. E‐I) Growth curve recorded from each mouse in all treatment groups. J) H&E, IHC, and IF staining of Ki‐67, vimentin, N‐cadherin, E‐cadherin, and cP104aa in xenografts. K‐L) In vivo imaging was performed after tail vein injection of OSRC‐2 cells transfected with the four plasmids into nude mice, with fluorescent quantitative statistics performed on representative images. M‐N) Macroscopic appearance of lung metastatic nodule (arrows) and quantification of mouse lung metastasis. O) Representative H&E images of lung tissue sections. (t‐tests were used for two groups) Scale bar: 100 µm. Statistical significance is indicated (*P<0.05, **p < 0.01, ***p < 0.001) by Student's t‐test or ANOVA.

To further validate these in vitro findings, the biological function of cP104aa was examined in vivo using xenograft tumor and lung metastasis models. OSRC‐2 cells transfected with the four vectors were injected subcutaneously into nude mice. IVIS imaging revealed stronger luciferase signals in tumors from the IRES‐mut2, OE‐cP104aa, and OE‐circPVT1 groups compared to the NC group (**Figure** **5**A,B). Tumor weights and volumes were significantly greater in these groups, indicating that cP104aa and circPVT1 overexpression promoted tumor growth (Figure 5C–I). IHC and immunofluorescence (IF) analyses of Ki‐67, E‐cadherin, N‐cadherin, vimentin, and cP104aa confirmed these results. Ki‐67 expression was higher in the IRES‐mut2, OE‐cP104aa, and OE‐circPVT1 groups than in the control group, and EMT marker trends mirrored those observed in vitro (Figure 5J). Additionally, lung metastasis models were constructed using the same cell lines. IVIS imaging showed that the IRES‐mut2, OE‐cP104aa, and OE‐circPVT1 groups exhibited increased luciferase signal strength and area compared to the control group (Figure 5K,L). Furthermore, the volume and number of pulmonary metastatic nodules were significantly higher in the IRES‐mut2, OE‐cP104aa, and OE‐circPVT1 groups (Figure 5M–O). Together, these assays provide strong evidence that circPVT1 and its encoded cP104aa peptide promote both tumor growth and metastasis.

### 2.5 cP104aa Interacts with HNRNPK, which Inhibits Ubiquitination of c‐MYC Through WWP2

**Figure 6:**
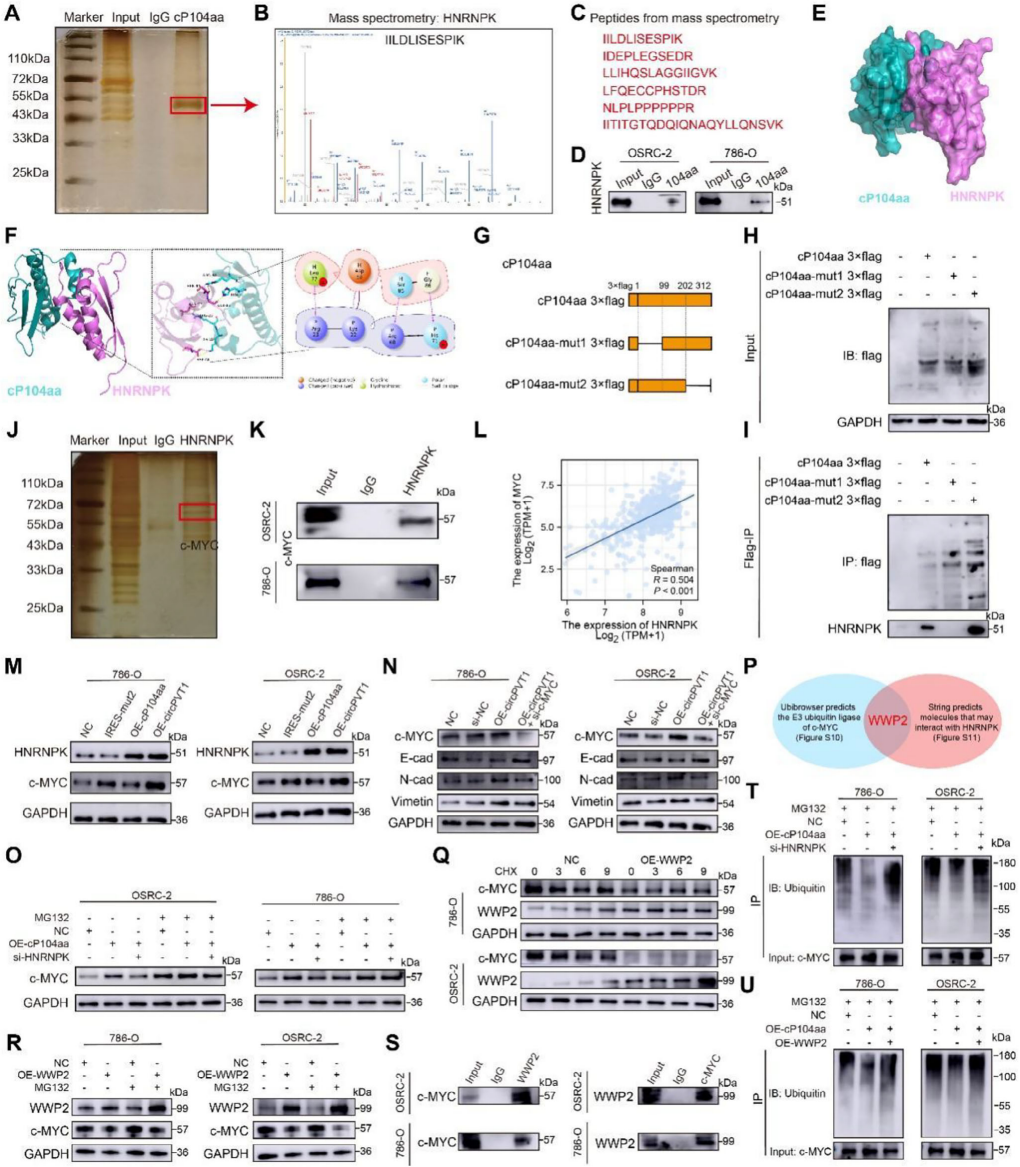
cP104aa interacts with HNRNPK, which inhibits ubiquitination of c‐MYC through WWP2. A–C) Co‐IP and silver staining show the molecular weight of cP104aa‐binding proteins, along with mass spectrometry analysis of the cP104aa‐enriched band. D) Co‐IP confirmed HNRNPK as a binding protein of cP104aa in OSRC‐2 and 786‐O cells. E) PyMOL software displayed the 3D protein structures of cP104aa and HNRNPK and their interaction. F) Details of the binding sites between cP104aa and HNRNPK identified using Maestro software. G) Schematic illustration of cP104aa expression plasmids. H‐I) Immunoblot analysis after transfection of indicated truncation mutants. J) Silver staining showing the molecular weight of HNRNPK‐binding proteins. K) Co‐IP confirmed c‐MYC as the binding protein of HNRNPK in OSRC‐2 and 786‐O cells. L) HNRNPK expression positively correlated with c‐MYC. M) Western blot analysis of HNRNPK and c‐MYC expression in the four groups. N) Western blot analysis of vimentin, N‐cadherin, and E‐cadherin in OSRC‐2 and 786‐O cells treated with OE‐circPVT1 and si‐c‐MYC. O) Protein level of c‐MYC in OSRC‐2 and 786‐O cells treated with 20 µM MG132 for 12 h after transfection with NC, OE‐cP104aa, and si‐HNRNPK. P) Venn diagram indicating the potential E3 ubiquitin ligase of c‐MYC that may interact with HNRNPK. Q) Western blot analysis of c‐MYC expression in control or OE‐WWP2‐treated cells with CHX. R) Western blot analysis of c‐MYC levels in control and OE‐WWP2 cells, with or without MG132 treatment. S) Co‐IP assays showing endogenous interaction between WWP2 and c‐MYC. T, U) Ubiquitination modification of c‐MYC proteins in OSRC‐2 and 786‐O cells, determined via Co‐IP and western blot after transfection with NC, OE‐cP104aa, and si‐HNRNPK or OE‐WWP2.

To elucidate the mechanism by which cP104aa functions in RCC cells, an IP assay was performed to identify potential binding proteins (**Figure** **6**A). LC–MS/MS analysis revealed that HNRNPK was co‐immunoprecipitated with cP104aa (Figure 6B,C), and this interaction was further validated by WB experiments (Figure 6D). Docking simulations using PyMOL and HDOCK predicted two binding sites between cP104aa and HNRNPK (Figure 6E,F). To assess their functional activity, two truncated cP104aa overexpression vectors with Flag tags were constructed (Figure 6G). Co‐IP assays confirmed that cP104aa‐mut1 facilitated the interaction between cP104aa and HNRNPK (Figure 6H,I). HNRNPK regulates c‐MYC expression,^[^ ^16^ ^]^ with c‐MYC playing a key role in the development of various tumors, including RCC, as an oncogenic factor.^[^ ^17^ ^]^ IP and silver staining assays revealed distinct enrichment of bands between 50–70 kDa (Figure 6J). WB analysis confirmed the direct interaction between HNRNPK and c‐MYC (Figure 6K). Furthermore, TCGA database analysis demonstrated a positive correlation between c‐MYC and HNRNPK expression in RCC (Figure 6L). Next, the expression levels of HNRNPK and c‐MYC were examined in the four experimental groups. HNRNPK expression was elevated in the cP104aa and OE‐circPVT1 groups, while c‐MYC levels were upregulated in the IRES‐mut2, cP104aa, and OE‐circPVT1 groups (Figure 6M). To investigate whether circPVT1 regulates the EMT pathway through c‐MYC, WB assays showed that c‐MYC knockdown reversed the upregulation of N‐cadherin and vimentin and the downregulation of E‐cadherin induced by circPVT1 overexpression (Figure 6N). WB assays further revealed that overexpression of cP104aa increased c‐MYC expression, while c‐MYC levels were reduced upon si‐HNRNPK treatment (Figure 6O). Given that c‐MYC is known to undergo ubiquitination,^[^ ^18^ ^]^ MG132 was used to assess the stability of the c‐MYC protein. The results showed that MG132 enhanced the upregulation of c‐MYC induced by cP104aa overexpression (Figure 6O). To identify potential interaction partners of HNRNPK, with a particular focus on E3 ubiquitin ligases of c‐MYC, the Ubibrowser (http://ubibrowser.bio‐it.cn/) (Figure S8, Supporting Information) and String (https://cn.string‐db.org/) (Figure S9, Supporting Information) were utilized. WWP2 was identified as the only E3 ligase of c‐MYC that may interact with HNRNPK (Figure 6P). CHX chase assays demonstrated that overexpression of WWP2 significantly accelerated c‐MYC degradation (Figure 6Q). WWP2 overexpression led to a decrease in c‐MYC protein levels in 786‐O and OSRC‐2 cells, an effect reversed by MG132 treatment (Figure 6R). Co‐IP experiments further confirmed the endogenous interaction between WWP2 and c‐MYC (Figure 6S). The binding between WWP2 and HNRNPK was also verified by Co‐IP, confirming their interaction (Figure S10, Supporting Information). To assess the impact on c‐MYC ubiquitination, Co‐IP and WB assays were conducted, suggesting that cP104aa overexpression significantly reduced c‐MYC ubiquitination levels. Moreover, the addition of si‐HNRNPK or OE‐WWP2 partially restored c‐MYC ubiquitination (Figure 6T,U). Collectively, these results suggest that cP104aa interacts with HNRNPK to upregulate c‐MYC expression by inhibiting WWP2‐mediated c‐MYC ubiquitination, thereby promoting RCC proliferation and metastasis.

### 2.6 CircPVT1 Regulates c‐MYC by Binding to EIF4A3

**Figure 7:**
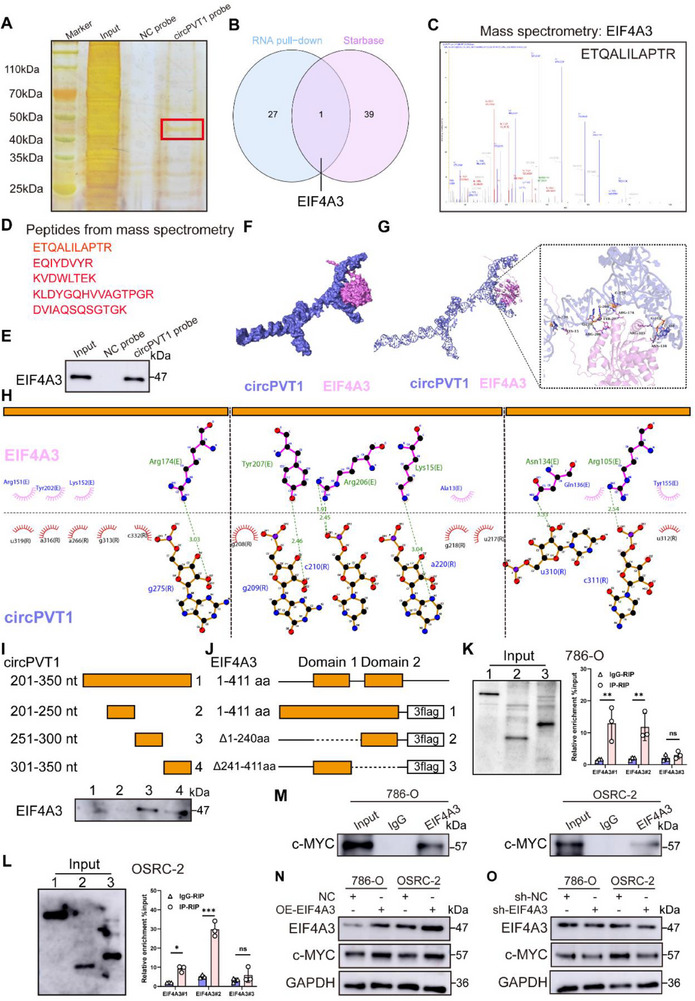
CircPVT1 promotes c‐MYC expression via binding with EIF4A3. A) RNA pull‐down assay using circPVT1 and NC probes. B‐D) Mass spectrometry analysis and Venn diagram showed circPVT1‐binding proteins identified using the RNA pull‐down assay. E) Western blotting was performed after RNA pull‐down assay. F‐H) PyMOL and Maestro software displayed the interaction and details of binding sites between cP104aa and HNRNPK in their 3D protein structures. I) RNA pull‐down with serial deletions of circPVT1 identified the essential regions on circPVT1 necessary for binding to EIF4A3. J) Schematic representation of full‐length and truncated EIF4A3 isoforms. K, L) Western blot verification that cells transfected with plasmids successfully expressed full‐length and truncated EIF4A3 with 3× Flag (left) and qRT‐PCR demonstrate that circAPVT1 binds to EIF4A3 amino acids 241–411 (right). M) Co‐IP confirmed c‐MYC as the binding protein of EIF4A3 in OSRC‐2 and 786‐O cells. N, O) Western blot analysis showed that EIF4A3 could regulate the expression of c‐MYC.

Previous functional experiments demonstrated that both circPVT1 and cP104aa significantly promote RCC cell proliferation and migration. However, the tumor‐promoting effect of OE‐circPVT1 was more pronounced than that of OE‐cP104aa. This suggests that circPVT1 may contribute to RCC progression through additional mechanisms. Given that circRNAs are known to interact with RNA‐binding proteins (RBPs) within ribonucleoprotein complexes,^[^ ^16^ ^]^ the present study investigated whether circPVT1 facilitates RCC tumorigenesis by binding to specific proteins. IP and silver staining assays revealed the enrichment of distinct bands between 40–50 kDa (Figure 7A). Mass spectrometry and starBase screening identified EIF4A3 as a potential binding partner for circPVT1 (Figure 7B–D). Further experiments confirmed the direct interaction between circPVT1 and EIF4A3 (Figure 7E). Docking simulations using PyMOL and HDOCK revealed three binding sites between circPVT1 and EIF4A3 (Figure 7F–H). To pinpoint the specific binding sites on circPVT1, truncated circPVT1 mutants were synthesized: mut1 (201–250 bp), mut2 (251–300 bp), and mut3 (301–351 bp). OSRC‐2 cells transfected with these mutants were subjected to a labeled RNA streptavidin pull‐down assay. WB analysis showed that EIF4A3 was present in the pull‐down fractions of cells transfected with either circPVT1 251–300 or 301–350 nt but absent in the fractions from circPVT1 201–250 transfected cells (Figure 7I). To further explore the binding sites of circPVT1 and EIF4A3, plasmids encoding truncated EIF4A3 isoforms were generated based on its structural domains.^[^ ^19^ ^]^ These plasmids were transfected into 786‐O and OSRC‐2 cells to express the corresponding proteins (Figure 7J,K,L, left). Then, RNA samples obtained by RIP experiments were amplified by qRT‐PCR, and the results showed that circPVT1 binds to amino acids 241–411 of EIF4A3 (Figure 7K,L, right). Since EIF4A3 is known to regulate c‐MYC expression,^[^ ^20^ ^]^ the relationship between EIF4A3 and c‐MYC was examined in RCC. Analysis revealed a positive correlation between c‐MYC and EIF4A3 expression (Figure S11, Supporting Information), and WB experiments confirmed the direct interaction between EIF4A3 and c‐MYC (Figure 7M). Furthermore, a positive correlation between c‐MYC and EIF4A3 expression was observed in RCC cells (Figure 7N,O). In summary, these results suggest that circPVT1 promotes c‐MYC expression by binding to EIF4A3, thus contributing to RCC progression.

### 2.7 Axitinib is a Potential cP104aa Antagonist

**Figure 8:**
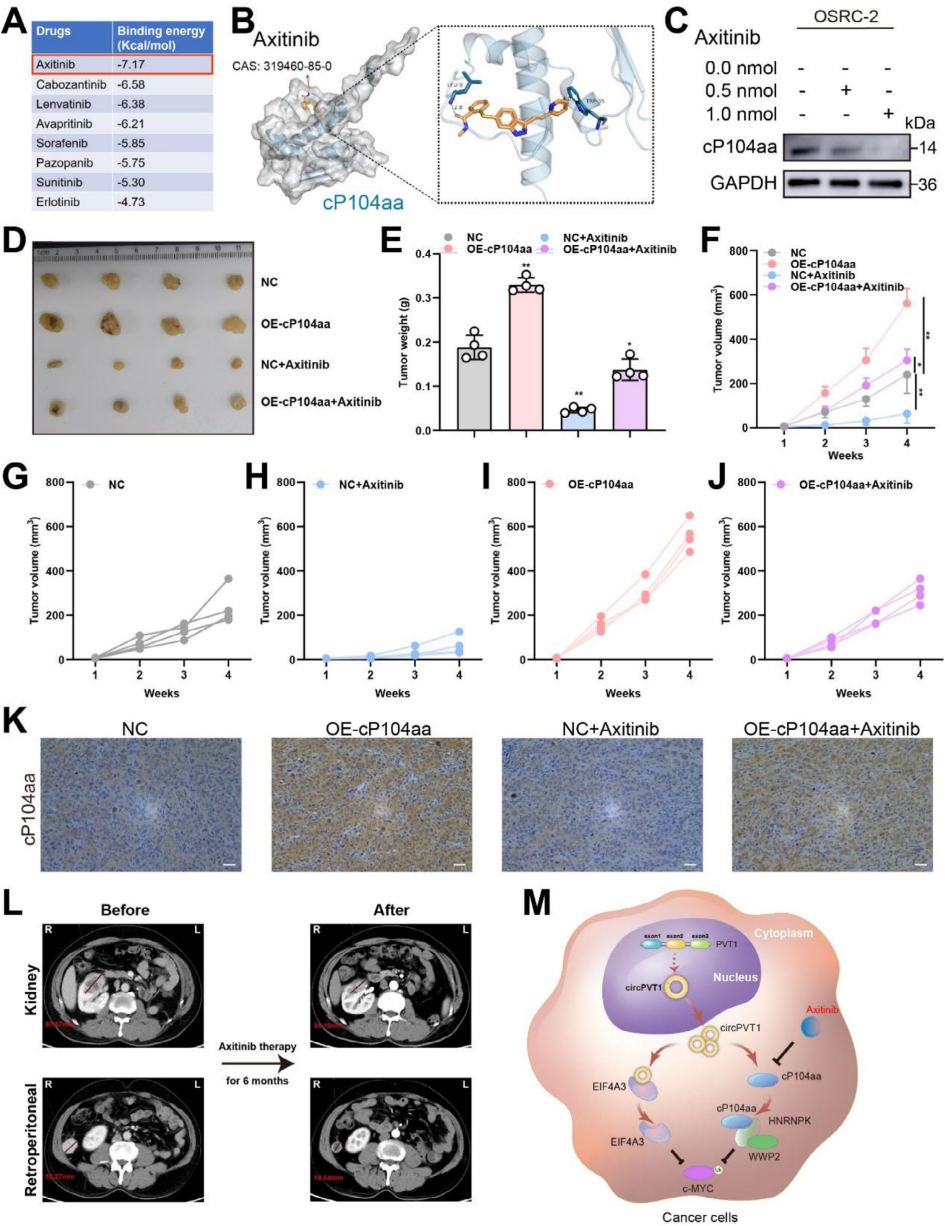
Axitinib is a potential cP104aa antagonist. A) Molecular docking results of cP104aa. B) Molecular docking interaction of compounds within the binding site of cP104aa. C) Changes in cP104aa expression with axitinib treatment. D‐E) Representative images of xenograft tumors in nude mice and the average tumor weight of nude mice. F‐J) Growth curve recorded from each mouse in all treatment groups. K) IHC of cP104aa in xenografts. L) CT images of the kidney before and after axitinib treatment. M) Proposed model for the potential function of circPVT1 and cP104aa in RCC progression. Scale bar: 100 µm. Statistical significance is indicated (*p < 0.05, **p < 0.01) by Student's t‐test or ANOVA.

Our experimental studies highlighted the critical role of cP104aa in tumor progression, prompting an investigation into potential antagonists. To identify suitable candidates for targeted treatment, PyMOL software was used to screen eight molecularly targeted drugs commonly employed in RCC therapy. Based on the interfacial interaction score, axitinib was selected for further investigation (**Figure** **8**A). The 3D structure of the interaction between cP104aa and axitinib was then explored through computational docking (Figure 8B). Subsequent experiments assessed the sensitivity of OSRC‐2 cell lines to axitinib, demonstrating that axitinib effectively inhibited cP104aa expression (Figure 8C). These findings were further validated in vivo, with or without axitinib treatment. In a subcutaneous tumor model, axitinib treatment at a dose of 30 mg kg^−1^ yielded significant therapeutic benefits (Figure 8D). Tumor progression induced by cP104aa overexpression was effectively reversed with axitinib treatment (Figure 8E–J), and IHC analysis confirmed the inhibition of cP104aa expression by axitinib (Figure 8K). Furthermore, the response to axitinib treatment was evaluated in a patient with high cP104aa expression. In November 2020, enhanced CT scans revealed a primary tumor in the right kidney with metastasis to the right retroperitoneum. After six months of axitinib treatment, follow‐up CT scans showed a reduction in the size of both tumors (Figure 8L). In summary, these data demonstrate the inhibitory effect of axitinib on cP104aa and suggest its potential as a key therapeutic agent for patients with high cP104aa expression.

## 3 Discussion

CircRNAs, with their unique structure and high expression levels, hold great potential as biomarkers for human diseases,^[^ ^21^ ^]^ and are critically involved in various conditions, including cancer.^[^ ^5^, ^22^ ^]^ Recently, circRNAs have been demonstrated to encode proteins.^[^ ^23^ ^]^ For example, circ‐ZNF609 contains an ORF capable of translation and plays a key role in myogenesis.^[^ ^24^ ^]^ Additionally, several protein‐coding circRNAs involved in cancer progression have been identified, such as the protein encoded by circPPP1R12A, which contributes to tumor pathogenesis in colon cancer.^[^ ^25^ ^]^ An increasing number of circRNAs have now been recognized for their ability to encode proteins.^[^ ^15^, ^26^ ^]^ These findings highlight the need for further exploration of the functional capabilities of circRNAs.

Research has increasingly emphasized the pivotal role of circPVT1 in the development and progression of various cancers. For instance, circPVT1 promotes cell growth in B‐cell lymphoma through a non‐miRNA‐binding mechanism.^[^ ^27^ ^]^ Furthermore, circPVT1 is abundantly present in exosomal vesicles and plays a significant role in the interaction between acute myeloid leukemia cells and the tumor microenvironment.^[^ ^28^ ^]^ The present study demonstrated that circPVT1 is upregulated in patients with RCC and correlates with increased tumor size and distal metastasis. Both in vitro functional experiments and in vivo xenograft assays revealed the central role of circPVT1 in RCC development and metastasis. Additionally, the mechanisms by which circPVT1 promotes tumor progression were investigated. The circRNADb database revealed that circPVT1 contains two IRES sequences and an ORF, suggesting its potential to encode a novel peptide, cP104aa, which was confirmed through LC–MS/MS. In some cases, the encoded peptides exert a biological effect independently,^[^ ^26b^ ^]^ while in others, both the peptide and circRNA collaborate in the biological process.^[^ ^29^ ^]^ Our results indicated that tumor cells in the OE‐circPVT1 group exhibited stronger growth and invasion capabilities than those in the OE‐cP104aa group, suggesting that both cP104aa and circPVT1 play critical roles in tumor progression. Consequently, the molecular mechanisms by which cP104aa and circPVT1 contribute to RCC pathogenesis were further explored.

C‐MYC, a member of a gene superfamily frequently activated in human cancers, is associated with poor patient survival.^[^ ^30^ ^]^ As a transcription factor, c‐MYC regulates the expression of various genes involved in numerous biological processes, both directly and indirectly.^[^ ^31^ ^]^ Its activation is integral to many aspects of cancer, including uncontrolled proliferation, metastasis, immune evasion, and metabolic reprogramming.^[^ ^32^ ^]^ Upregulation of c‐MYC in tumors is often driven by genomic alterations such as gene amplification or post‐translational modifications.^[^ ^33^ ^]^ The present study identified a novel mechanism by which cP104aa and circPVT1 regulate c‐MYC expression in RCC. Our findings demonstrated that circPVT1 promotes tumor progression through dual mechanisms: cP104aa increases c‐MYC expression by forming a complex with HNRNPK, while circPVT1 enhances c‐MYC expression by binding to the EIF4A3 protein.

The critical role of cP104aa in RCC progression was confirmed through these analyses, emphasizing the importance of developing drugs that inhibit cP104aa expression. To personalize treatment responses, eight molecularly targeted drugs used in RCC therapy were screened. Axitinib, a tyrosine kinase inhibitor (TKI), was identified as effectively inhibiting cP104aa expression. Despite the limited number of biomarkers predicting targeted drug efficacy in RCC, our study is the first to show that cP104aa is a direct target of axitinib and that axitinib demonstrates significant anti‐cancer activity both in vitro and in vivo. These findings suggest that patients with high cP104aa expression may benefit from a more favorable therapeutic response to axitinib.

In conclusion, our findings highlight the critical role of cP104aa, a newly discovered peptide, and circPVT1 in RCC. The tumor‐promoting effects of circPVT1 were shown to be mediated through the upregulation of c‐MYC via dual mechanisms. Moreover, axitinib was identified as an innovative therapeutic approach, with potential pharmacological inhibition of cP104aa. These results are expected to provide valuable insights into the individualized treatment of RCC (Figure 8M).

## 4 Experimental Section

### Clinical Tissues

A total of 45 pairs of RCC specimens and adjacent normal tissues were collected from patients with RCC who underwent nephrectomy at Zhongda Hospital, Southeast University (Nanjing, China) between January 2019 and December 2020. After surgical resection, the tissues were immediately frozen and stored at −80 °C. The study protocol was approved by the Ethics Committees of Zhongda Hospital, Southeast University (2024ZDSYLL466‐P01). Written informed consent was obtained from all enrolled patients or their relatives.

### Cell Culture and Lentivirus Transfection

Human RCC cell lines (OSRC‐2, 786‐O, ACHN, and Caki‐2) and normal renal tubular epithelial cells (HK‐2) were obtained from the Cell Bank of the Chinese Academy of Sciences (Shanghai, China). HK‐2 cells were cultured in keratinocyte medium (KM; ScienCell, USA), while OSRC‐2, 786‐O, and Caki‐1 cells were cultured in Dulbecco's modified Eagle's medium (DMEM; Gibco, USA), and ACHN cells were cultured in RPMI‐1640 medium (Gibco). All media were supplemented with 10% fetal bovine serum (FBS; Hyclone, USA) and 1% penicillin/streptomycin (P/S; Yeasen, China). The circPVT1 overexpression vector, sh‐RNA against circPVT1 (sh‐circPVT1), negative control (NC) vector, IRES‐mut2, OE‐cP104aa, si‐HNRNPK, and OE‐WWP2 were synthesized by IBSBIO Company (Shanghai, China) (shRNA‐targeting sequence: AGCTCCCTCTAAAATGTCTGA (sh‐circPVT1#1); GCTGGGCTTGAGGCCTGATCT (sh‐circPVT1#2); CAGCTGGGCTTGAGGCCTGAT (sh‐circPVT1#3)). Transfection was performed according to the manufacturer's instructions, followed by puromycin selection (Gibco). The cell lines were stored at −80 °C using CELLSAVING reagent (NCM, Suzhou, China).

### Western Blotting

Proteins were extracted using RIPA lysis buffer (Beyotime, China) with protease inhibitors. Protein concentration was determined, and 30 µg of protein was separated via 10% and 12.5% SDS‐PAGE electrophoresis and then transferred to PVDF membranes. The membranes were blocked with 5% skim milk and incubated overnight with primary antibodies at 4 °C. Afterward, the membranes were incubated with secondary antibodies and visualized using ECL (NCM, Suzhou, China).

### Quantitative Real‐Time PCR, RNase R, MG132 Treatment, and Actinomycin D Assays

Total RNA from cells and tissues was extracted using the Total RNA Isolation Kit V2 (Vazyme Biotech Co., Ltd., China) with a tissue grinder (Jingxin, Shanghai). RNA concentration was measured with NanoDrop 2000, and relative expression was calculated using the 2^−ΔΔCt^ method. CircPVT1 high and low groups were categorized based on median expression. The OSRC‐2 and 786‐O cell lines were treated with 4 U mg^−1^ of RNase R (Epicentre Technologies, USA) and incubated for 30 min at 37 °C. RNA was reverse transcribed with specific primers and quantified using qRT‐PCR. For protein degradation analysis, cells were treated with 20 µM MG132 (MedChemExpress, USA) for 12 h. Protein levels and ubiquitination were assessed by Co‐IP and WB.

To block transcription, the OSRC‐2 and 786‐O cell lines were treated with actinomycin D (2 mg mL^−1^, Merck, Germany), and RNA from the treated cells was extracted for qRT‐PCR analysis.

### Subcellular Fractionation

RNA isolation from nuclear and cytoplasmic fractions was conducted using the RNA Subcellular Isolation Kit (Thermo, USA). U6 served as the nuclear control, while GAPDH was used as the cytoplasmic control.

### cP104aa Antibody Preparation

Following the manufacturer's instructions (Yuanpeptide Biotechnology Co., Ltd, Nanjing, China), the main steps involved antigen preparation, animal immunization, blood sampling, antibody extraction and purification, and antibody specificity and titer testing. In the antigen preparation phase, bacterial cultures were grown at 37 °C for 4 h, then collected via centrifugation. The bacteria were lysed with buffer (PBS pH 7.5 + 10% glycerol), and the soluble supernatant contained native proteins (NPE). The insoluble portion was solubilized using a denaturing buffer (urea 8 m), and after centrifugation, the supernatant contained denatured proteins (DPE). The extracted DPE was used for multiple immunizations of rabbits, after which serum was collected, and antibodies were extracted and purified. Finally, antibody specificity and titer were tested.

### Fluorescence In Situ Hybridization (FISH)

For FISH, OSRC‐2 and 786‐O cells were cultured on coverslips, fixed, and permeabilized. After pre‐hybridization, the cells were incubated overnight at 37 °C in the dark with a Cy‐3‐labeled circPVT1‐specific probe (RiboBio, Guangzhou, China). The cells were then processed as previously described,^[^ ^34^ ^]^ and images were captured using a confocal microscope.

### EdU and Colony Formation Assay

EdU assays were performed by incubating the cells with 10 µM EdU (Beyotime). The cells were fixed with 4% paraformaldehyde and blocked with PBS/0.3% bovine serum albumin (BSA). The cells were subsequently incubated with Alexa Fluor 555 and DAPI in the dark. EdU incorporation was visualized using a Leica DM6 B upright microscope system (Leica, Germany). For the colony formation assay, 5 × 10^2^ cells were seeded into 6‐well plates. After 2 weeks, the colonies were stained and photographed.

### Colloidal Coomassie Staining

Cell lysates were subjected to electrophoresis, and an appropriate amount of Coomassie staining solution (Beyotime, China) was added. The gel was shaken at room temperature for 30 min until the target protein bands were visible. The staining solution was then discarded, and the gel was washed thoroughly before being documented.

### Wound Healing and Transwell Assays

Cell migration was assessed using the wound healing assay. A wound was created using a 200 µL tip along the 6‐well plate, and the results were recorded at 0 and 24 h. For the transwell assay, invasion ability was assessed by adding 100 µL of matrix (BD Biosciences, USA) to the upper chamber. RCC cells (3 × 10^5^) were resuspended in the upper chambers without FBS, and 700 µL of medium containing 10% FBS was added to the bottom chambers. After incubating for 24 h, the cells on the lower surface of the membrane were stained with 0.1% crystal violet and photographed.

### RNA Pull‐Down Assay

The probes (Genepharma, China) were incubated with the lysate for 24 h at room temperature. Streptavidin magnetic beads were then mixed with the lysate and incubated for 2 h. After incubation, the magnetic beads were collected and washed three times. The beads were rinsed and subsequently prepared for silver‐stained SDS‐PAGE and mass spectrometry analysis.

### Co‐Immunoprecipitation Assay

Lysis buffer containing a protease inhibitor cocktail was added to RCC cells at 4 °C. The primary antibody was incubated with protein A/G Sepharose beads (Beyotime). The protein was then incubated overnight with the primary antibody. The immunoprecipitation complexes were washed with IP buffer and analyzed via WB.

### Dual‐Luciferase Reporter Assay

The IRES‐mut1 and IRES‐mut2 sequences were inserted into the luciferase reporter gene of the circPVT1 plasmid (IBSBIO). Luciferase activity was measured using the Dual‐Luciferase Reporter Assay kit (Yeasen). The pmirGLO‐cP104aa luciferase reporter was constructed by IBSBIO (Shanghai, China). Promoter activity of cP104aa in cells was measured by luciferase assay according to previous literature.^[^ ^35^ ^]^ The translation efficiency of cP104aa is defined as the quotient of reporter protein production (F‐luc/R‐luc) divided by mRNA abundance.

### Immunohistochemistry (IHC)

Fresh samples were fixed in 4% paraformaldehyde and embedded in paraffin. The samples were sectioned into 5 µm slides and incubated with primary antibodies overnight at 4 °C. Afterward, the slides were incubated with secondary antibodies and photographed using a Leica microscope (Leica Microsystems, Germany).

### Immunofluorescence (IF)

RCC cells were fixed with BSA, permeabilized with 0.1% Triton X‐100, and incubated with the primary antibody overnight at 4 °C. After incubation with the secondary antibody, the stained cells were observed using a confocal laser scanning microscope (Zeiss, Germany).

### Mouse Xenograft and Pulmonary Metastasis Model

Four‐week‐old male BALB/c nude mice were obtained from the SLAC Laboratory Animal Company and randomly assigned to different groups. For the xenograft model, 1 × 10^6^ OSRC‐2 cells were subcutaneously injected into the mice. Tumor volume was measured and calculated weekly. In the lung metastasis model, 1 × 10^6^ cells in 100 µL were injected into the tail vein of the mice. After 4 weeks, metastases were monitored using an in vivo imaging system (AniView100, BLT, Guangzhou, China). The mice were then euthanized, and bilateral lung tissues were photographed. For axitinib treatment, OSRC‐2 cells were injected into the flanks of nude mice, followed by oral administration of axitinib (30 mg kg^−1^ dose^−1^) three times a week for 2 weeks.

### Statistical Analysis

Statistical analyses were performed using SPSS 23.0 software (IBM, USA). Student's t‐test and one‐way analysis of variance (ANOVA) were used for comparisons between two and multiple groups, respectively. Statistical significance was indicated by *p*‐values: *p < 0.05, **p < 0.01, and ***p < 0.001.

## Conflict of Interest

The authors declare that they have no competing interests.

## Author Contributions

H.‐L.Z. and T.T. contributed equally to this work. W.‐P.M., M.C., and B.X. designed the research. H.‐L.Z., T.T., J.J., T.‐L.Z., S.S., L.‐J.Z., J.‐P.W., and S.‐Q.C. performed the research and analyzed results. H.‐L.Z. and W.‐P.M. wrote the paper. All authors read and approved the final manuscript. H.Z. and T.T. contributed equally to this work.

## Supporting information



Supporting Information

Supplementary Data

## Data Availability

The data that support the findings of this study are available from the corresponding author upon reasonable request.
