# Supplementary material for: CircPVT1 Promotes Lung Metastasis and Tumor Progression in Renal Cell Carcinoma by Encoding the cP104aa Peptide and Targeting EIF4A3
Source: Adv Sci (Weinh). 2025 Sep 18;12(48):e01211. doi: 10.1002/advs.202501211 (PMC12752572; doi:10.1002/advs.202501211)
Supplement: Supplementary file 1 — Supporting Information [file ADVS-12-e01211-s001.docx]

**CircPVT1 promotes lung metastasis and tumor progression in renal cell carcinoma by encoding the cP104aa peptide and targeting EIF4A3**

Houliang Zhang, Tao Tao, Jie Ji, Tonglei Zhao, Si Sun, Lijie Zhang, Jianping Wu, Ming Chen, Shuqiu Chen, Bin Xu, Weipu Mao

**Supplementary Information**

[Supplementary Table 1. Antibody list - 3 -](#_Toc205054676)

[Supplementary Table 2. Primer sequences - 3 -](#_Toc205054677)

[Figure S1. circPVT1 expression in RCC cells after transfection. - 4 -](#_Toc205054678)

[Figure S2. Effects of circPVT1 knockdown *in vivo*. - 5 -](#_Toc205054679)

[Figure S3. Effects of circPVT1 overexpression on RCC cells. - 6 -](#_Toc205054680)

[Figure S4. CircPVT1 overexpression promotes RCC growth *in vivo*. - 7 -](#_Toc205054681)

[Figure S5. CircPVT1 overexpression promotes RCC lung metastasis *in vivo*. - 8 -](#_Toc205054682)

[Figure S6. Verification of translation efficiency of cP104aa by luciferase assay in vitro. - 9 -](#_Toc205054683)

[Figure S7. Transwell invasion assay was performed to detect the effects of four aforementioned plasmids on the invasion of OSRC-2 and 786-O cells. - 10 -](#_Toc205054684)

[Figure S8. Potential E3 ubiquitin ligases of c-Myc were predicted using Ubibrowser. - 11 -](#_Toc205054685)

[Figure S9. String predicts molecules that may interact with HNRNPK. - 12 -](#_Toc205054686)

[Figure S10. Co-IP assays showed endogenous interaction between WWP2 and HNRNPK. - 12 -](#_Toc205054687)

[Figure S11. Expression of c-MYC is positively related to EIF4A3 expression. - 13 -](#_Toc205054688)

# Supplementary Table 1. Antibody list

| Antibody Name | Manufacturer | Catalog Number | Application |
| --- | --- | --- | --- |
| anti-GAPDH | Abcam | ab181602 | WB (1:10000) |
| anti-cP104aa | yuanpeptide | 2200117A | WB (1:500), IHC (1:100) |
| anti-Bcl-2 | Abcam | ab182858 | WB (1:1000) |
| anti-E-Cadherin | Proteintech | 20874-1-AP | WB (1:5000), IF (1:200) |
| anti-N-Cadherin | Abcam | ab245117 | WB (1:1000), IF (1:200) |
| anti-Vimentin | Abcam | ab92547 | WB (1:1000), IF (1:200) |
| anti-c-MYC | Abcam | ab32072 | WB (1:1000), IHC (1:200) |
| anti-HNRNPK | ABclonal | A0772 | WB (1:1000), IHC (1:200) |
| anti-EIF4A3 | ABclonal | A8985 | WB (1:1000), IHC (1:200) |
| anti-DDDDK-Tag | ABclonal | AE005 | WB (1:1000) |
| anti-Bax | Abcam | ab32503 | IHC (1:200) |
| anti-Ki67 | Proteintech | 27309-1-AP | IHC (1:1000) |
| Anti-WWP2 | ABclonal | A2425 | WB (1:1000) |
| Ubiquitin | Cell signaling technology | 3936 | WB (1:1000) |

# Supplementary Table 2. Primer sequences

| Gene name | Primer sequences | Application |
| --- | --- | --- |
| GAPDH | F: 5’ -GGAGCGAGATCCCTCCAAAAT-3’  R: 5’ -GGCTGTTGTCATACTTCTCATGG -3’ | qRT-PCR |
| PVT1 | F: 5’-CCTGTGACCTGTGGAGACAC-3’  R: 5’-GTCCGTCCAGAGTGCTGAAA-3’ | qRT-PCR |
| CircPVT1 | F: 5’-GGTTCCACCAGCGTTATTC-3’  R: 5’-CAACTTCCTTTGGGTCTCC-3’ | qRT-PCR |

**
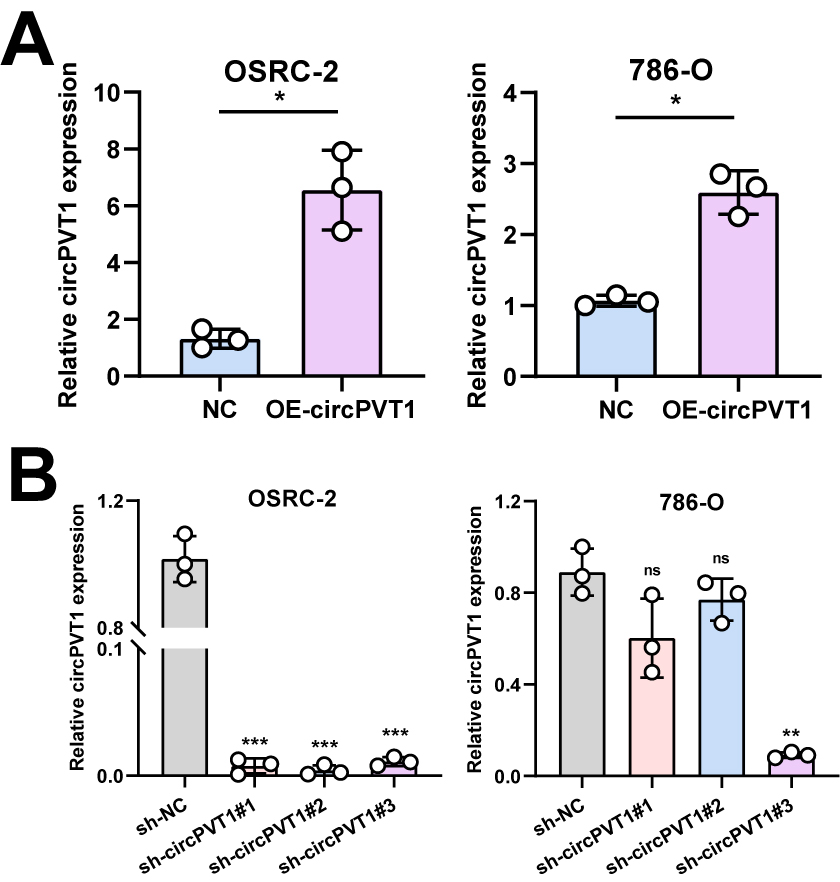
**

# Figure S1. circPVT1 expression in RCC cells after transfection.

**A)** Transfection of NC and OE-circPVT1. **B)** Transfection of sh-NC, sh-circPVT1#1, sh-circPVT1#3 and sh-circPVT1#3. N.S.: not significant. Statistical significance is indicated (*P<0.05, **P<0.01, ***P<0.001) by Student's t-test or ANOVA.

**
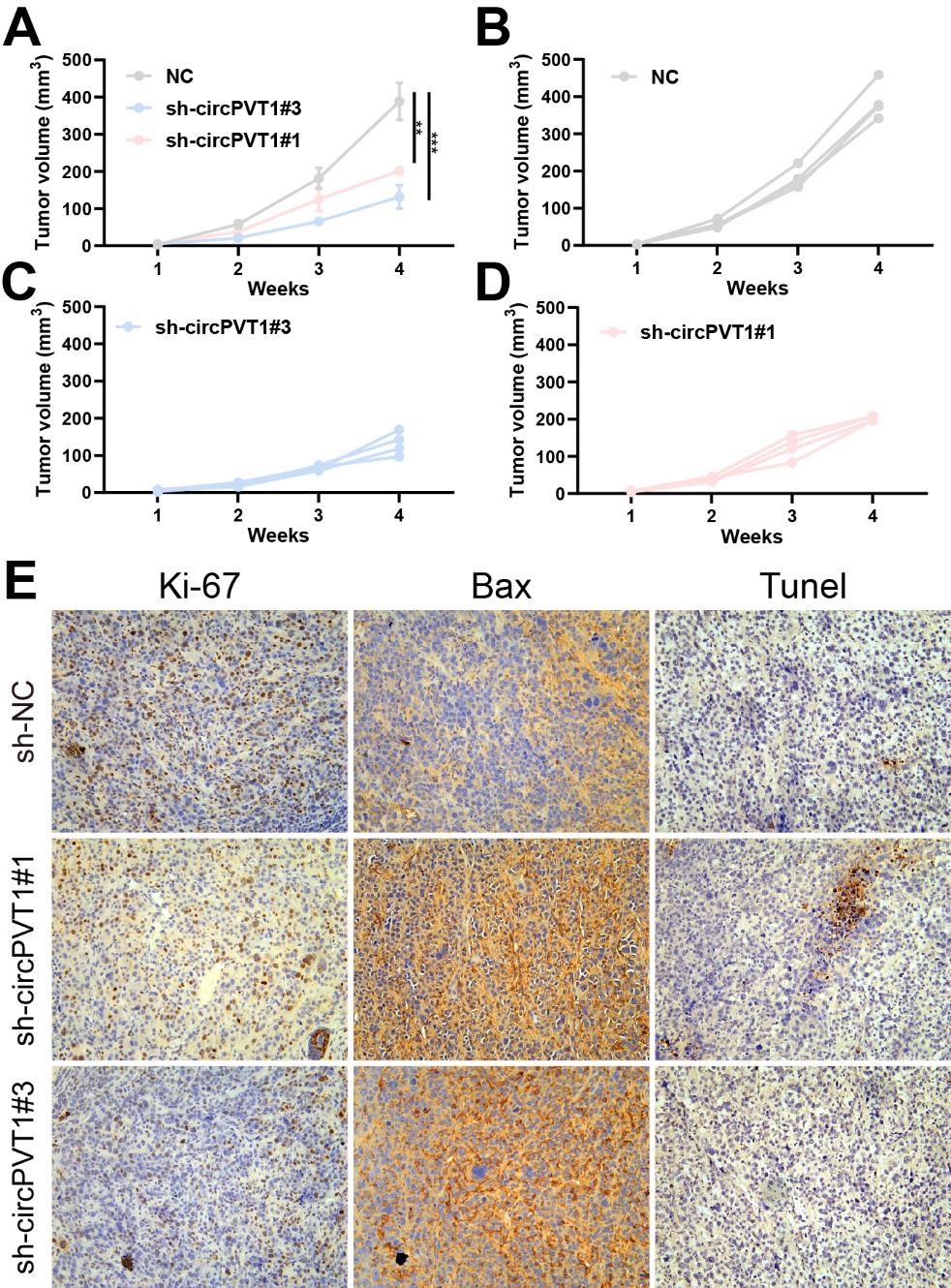
**

# Figure S2. Effects of circPVT1 knockdown *in vivo*.

**A-D)** Growth curves of xenografts. **E)** IHC of Ki-67, Bax, and Tunel in xenografts. Scale bar: 100 μm. Statistical significance is indicated (***P<0.001) by Student's t-test or ANOVA.

**
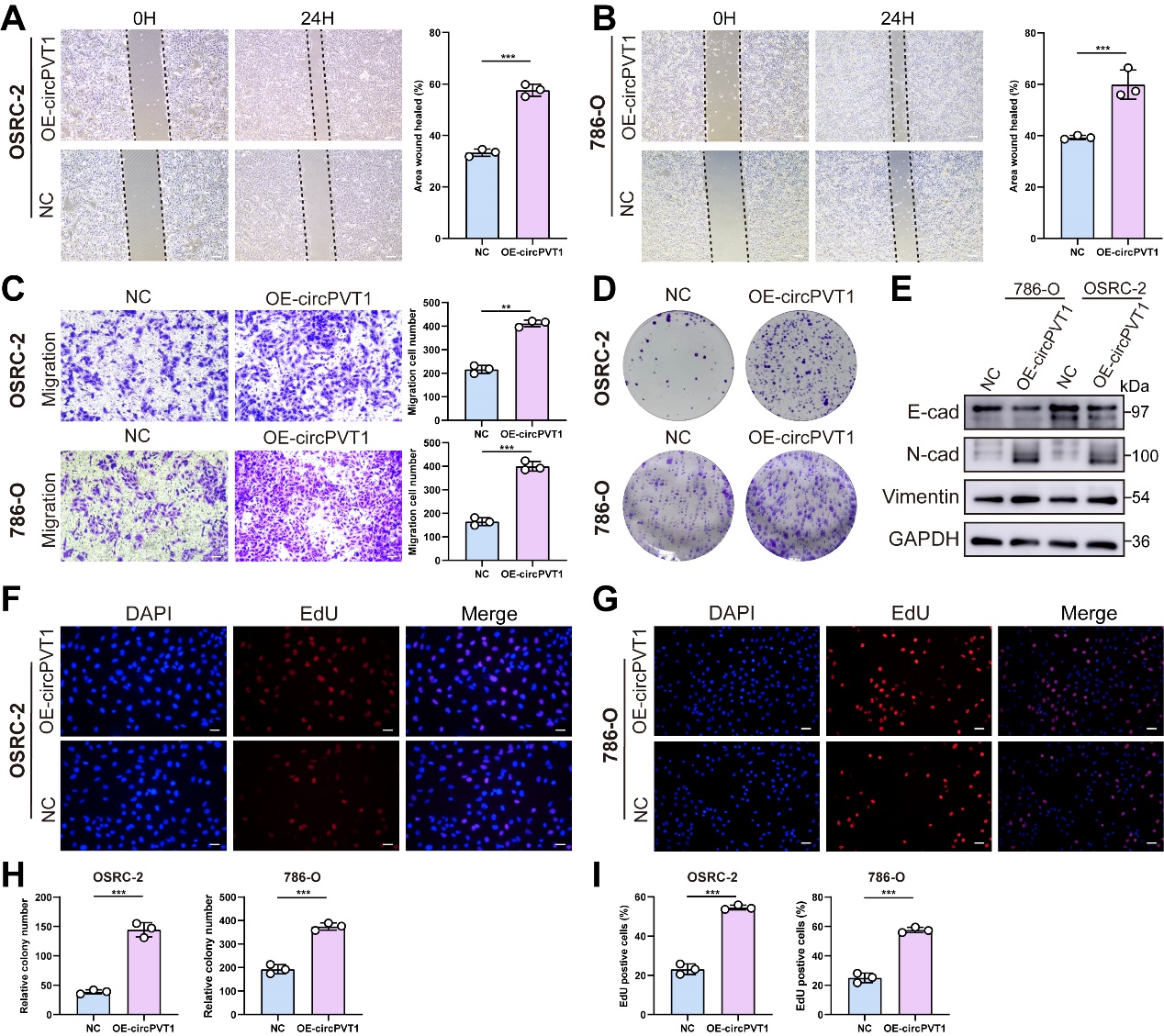
**

# Figure S3. Effects of circPVT1 overexpression on RCC cells.

**A, B)** Wound healing assay was conducted to detect the effect of circPVT1 overexpression on the migration of OSRC-2 and 786-O cells. **C**) Transwell migration assay was performed to detect the effects of circPVT1 overexpression on the migration of OSRC-2 and 786-O cells. **D)** Colony formation assay was performed to examine the effects of circPVT1 overexpression on proliferation in OSRC-2 and 786-O cells. **E)** Western blotting of vimentin, N-cadherin, and E-cadherin in OSRC-2 and 786-O cells treated with circPVT1 overexpression or negative control. **F, G)** EdU assay was conducted to determine the effects of circPVT1 overexpression on proliferation in OSRC-2 and 786-O cells. **H)** Colony numbers were calculated. **I)** Number of EdU-positive cells was counted in three randomly selected fields. Scale bar: 100 μm. Statistical significance is indicated (**P<0.01, ***P<0.001) by Student's t-test or ANOVA.

**
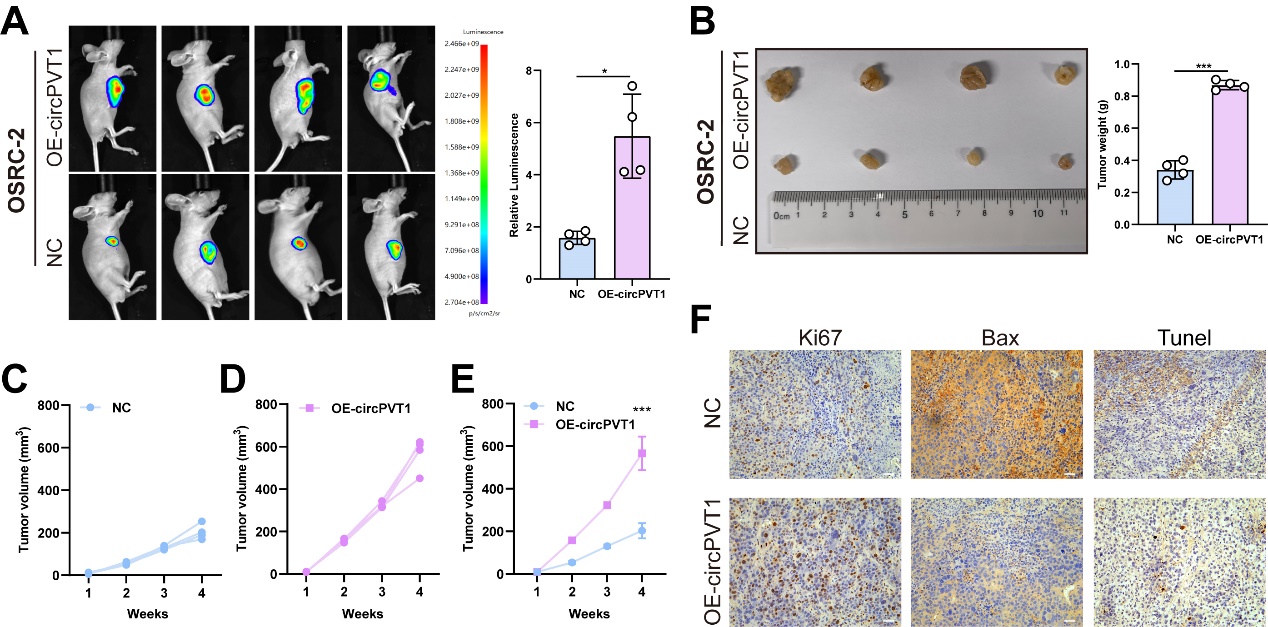
**

# Figure S4. CircPVT1 overexpression promotes RCC growth *in vivo*.

**A)** In vivo imaging was performed after injection of OSRC-2 cells overexpressing circPVT1 into nude mice, with fluorescent quantitative statistics performed on representative images. **B)** Tumors were photographed and weighed. **C-E)** Growth curves of xenografts. **F)** IHC of Ki-67, Bax, and Tunel in xenografts. Scale bar: 100 μm. Statistical significance is indicated (*P<0.05, ***P<0.001) by Student's t-test or ANOVA.

**
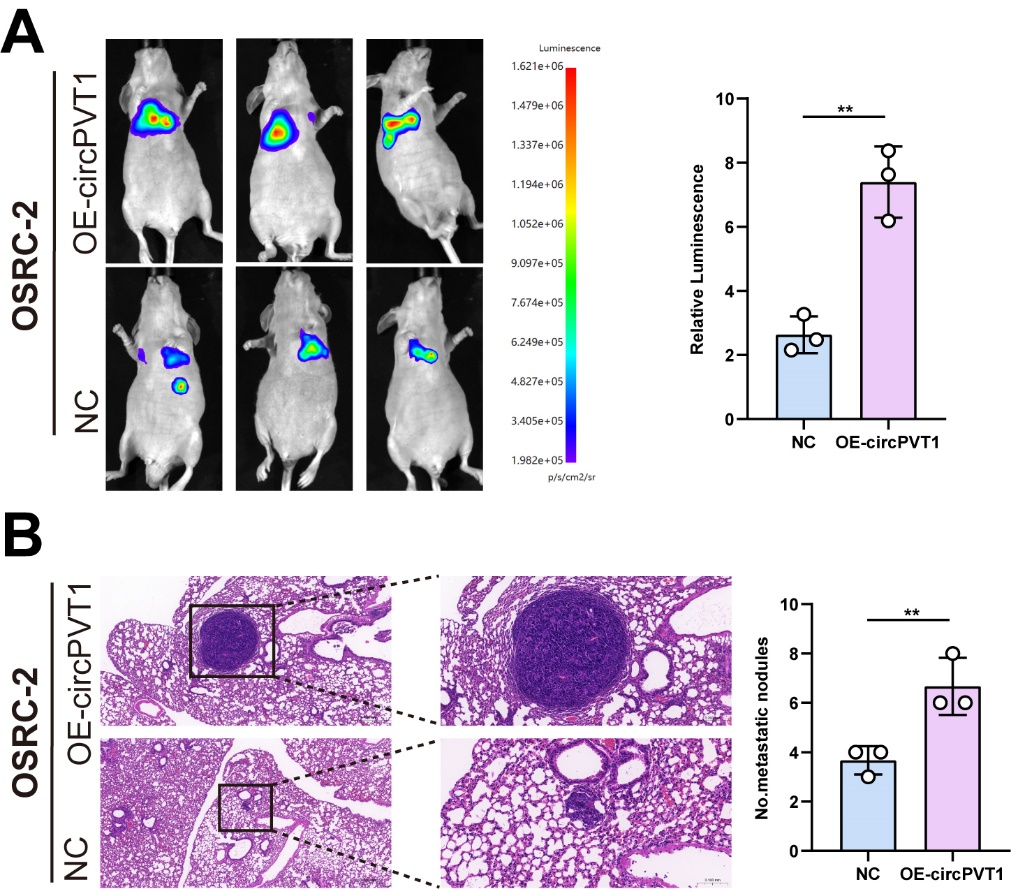
**

# Figure S5. CircPVT1 overexpression promotes RCC lung metastasis *in vivo*.

**A)** In vivo imaging was performed after tail vein injection of OSRC-2 cells overexpressing circPVT1 into nude mice and fluorescent quantitative statistics performed on representative images. **B)** H&E staining and quantification of mouse lung metastasis. Statistical significance is indicated (**P<0.01) by Student's t-test or ANOVA.


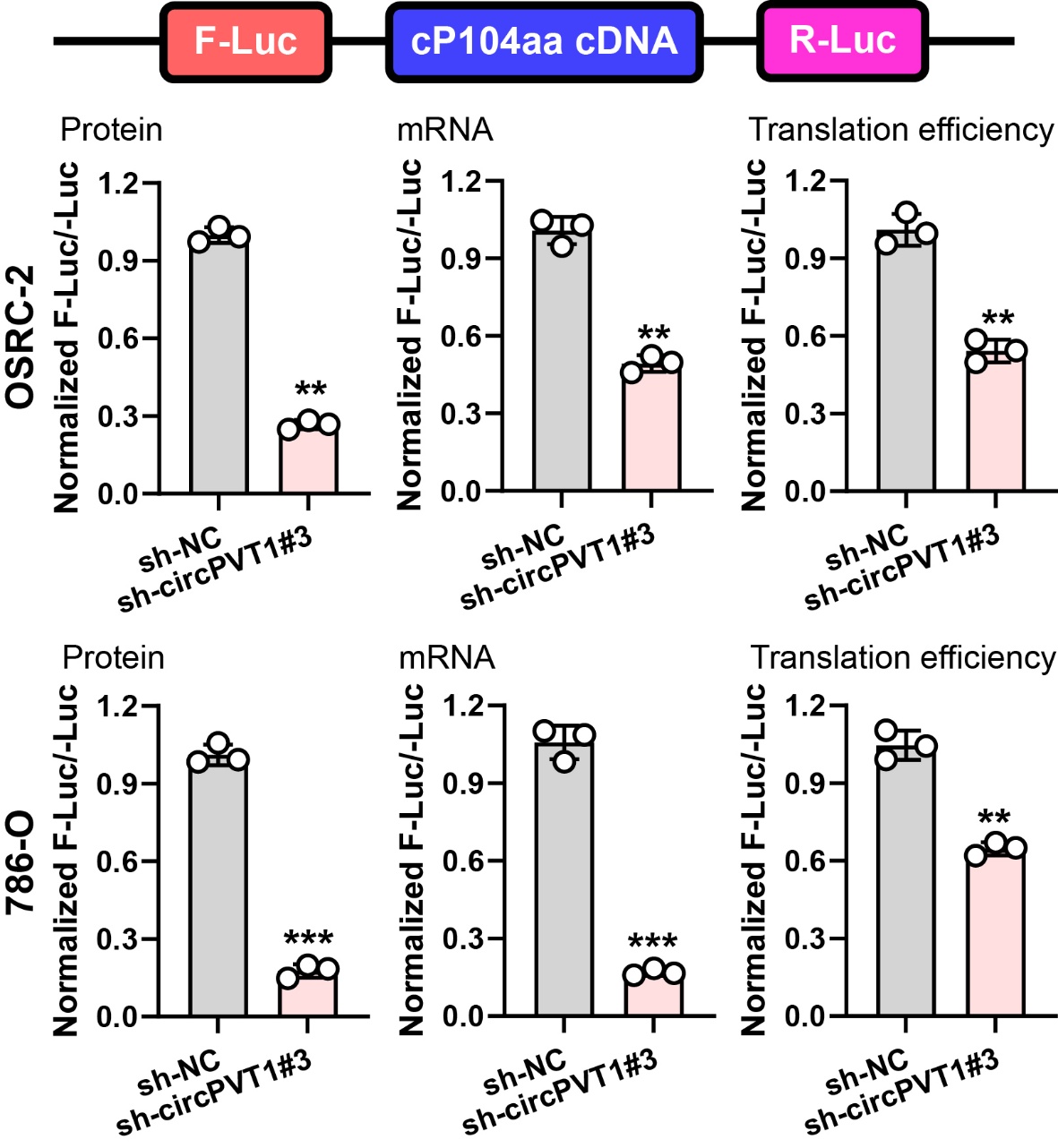


# Figure S6. Verification of translation efficiency of cp104aa by luciferase assay in vitro.

Wild-type or sh-circPVT1#3 OSRC-2 and 786-O cells were transfected with pmirGLO-cP104aa reporter for 24 h. The translation outcome was determined as a relative signal of F-luc divided by R-luc, the mRNA abundance was determined by qRT-PCR of F-luc and R-luc, and the translation efficiency of cp104aa is defined as the quotient of reporter protein production (F-luc/R-luc) divided by mRNA abundance.


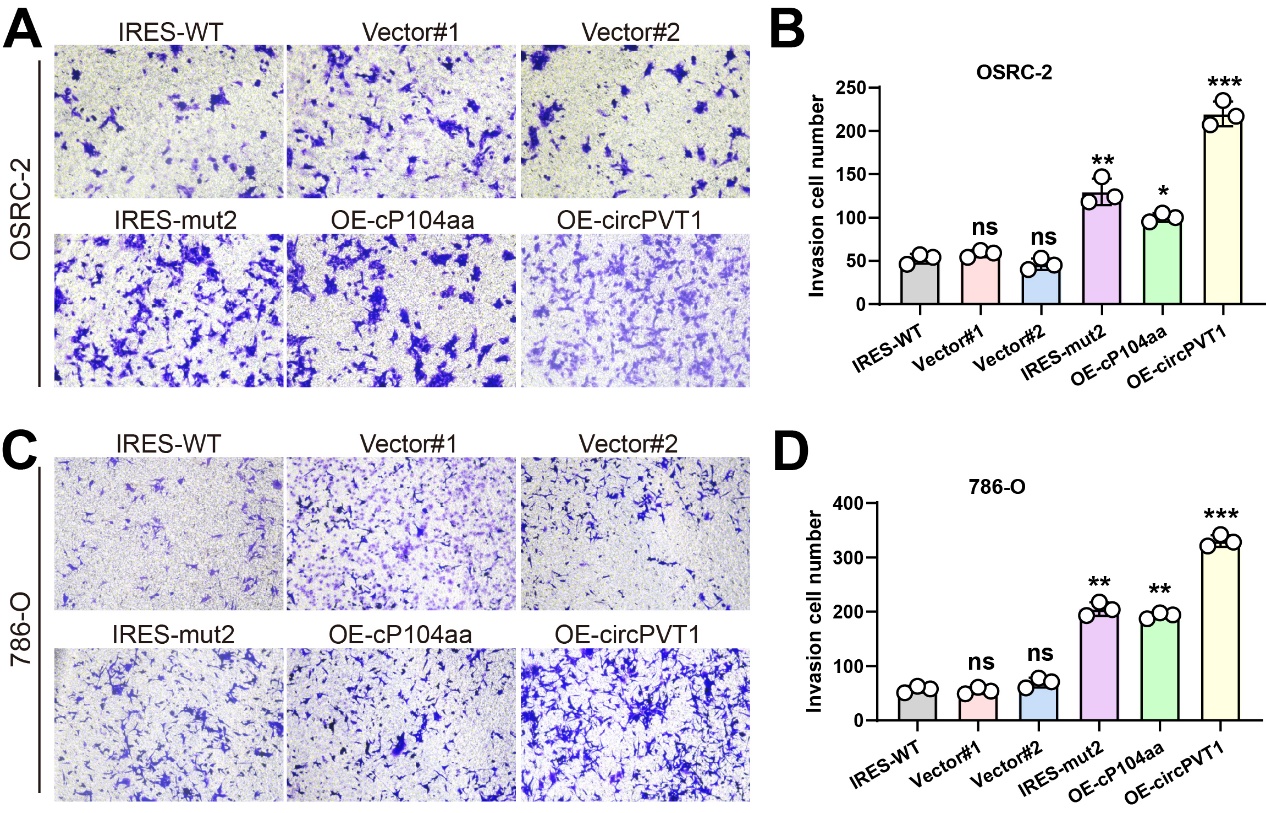


# Figure S7. Transwell invasion assay was performed to detect the effects of four aforementioned plasmids on the invasion of OSRC-2 and 786-O cells.

**A, B)** OSRC-2 cells. **C, D**) 786-O cells. Scale bar: 100 μm. Statistical significance is indicated (*P<0.05, ***P<0.001) by Student's t-test or ANOVA.

**
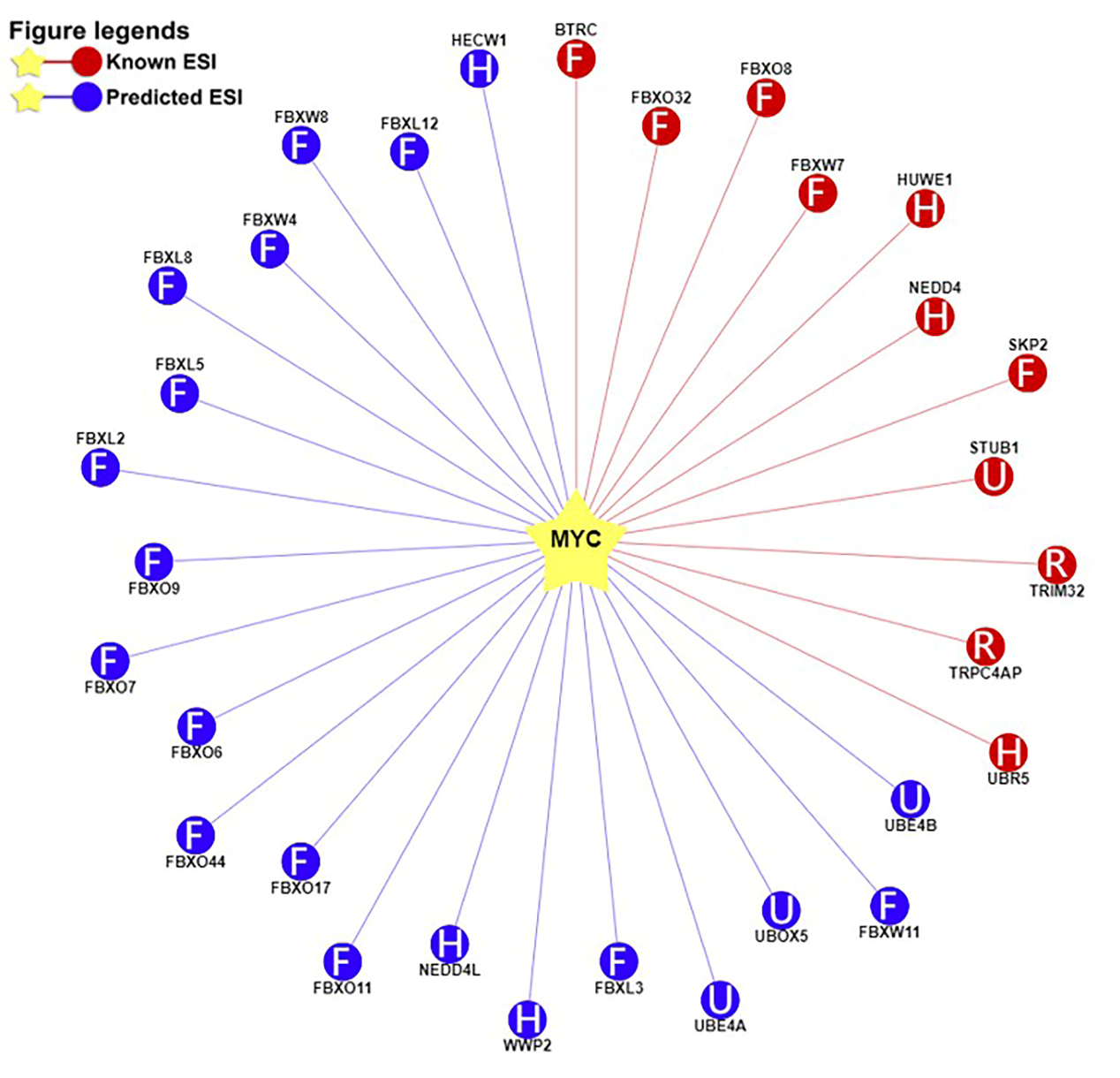
**

# Figure S8. Potential E3 ubiquitin ligases of c-Myc were predicted using Ubibrowser.

**
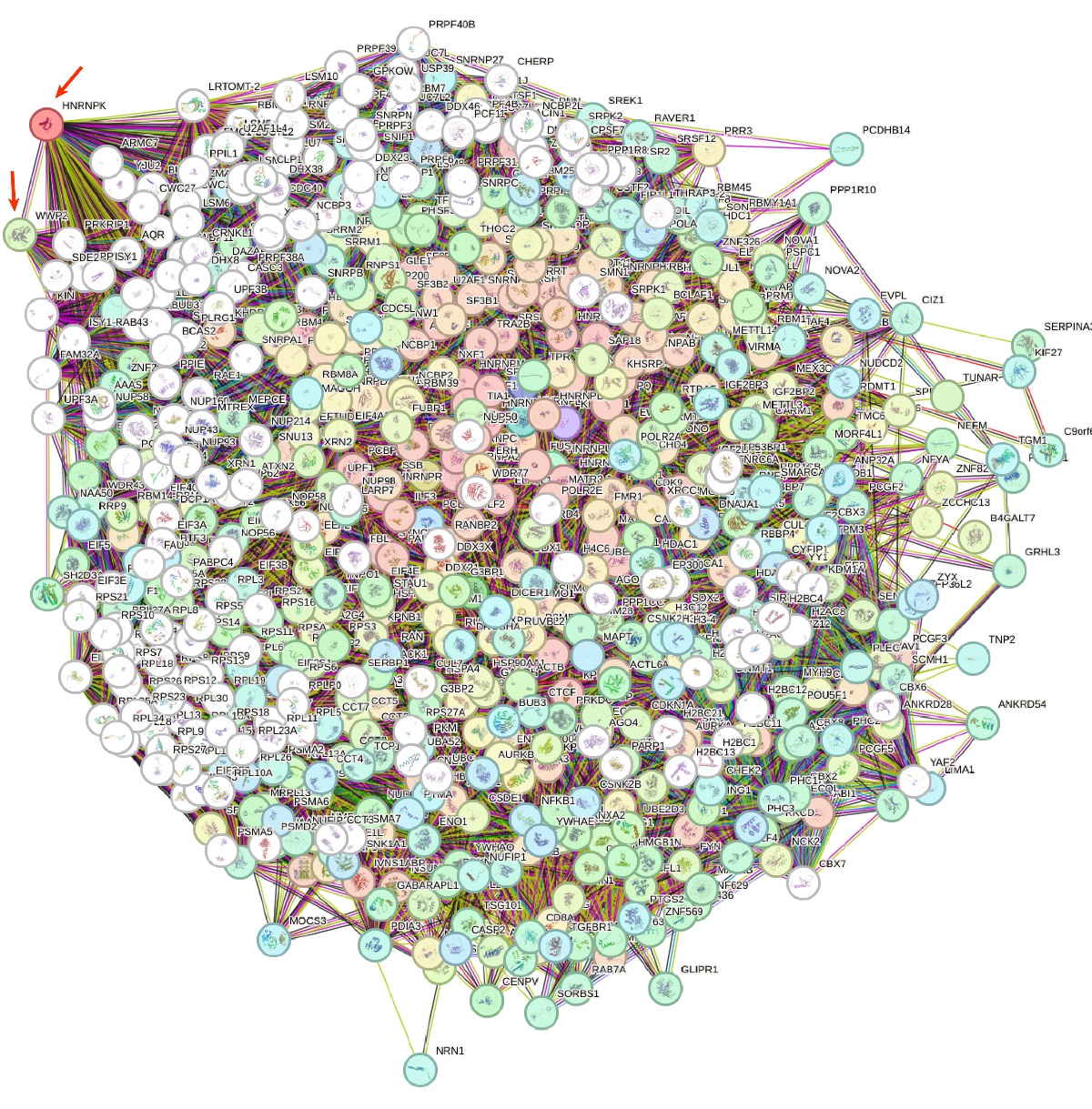
**

# Figure S9. String predicts molecules that may interact with HNRNPK.


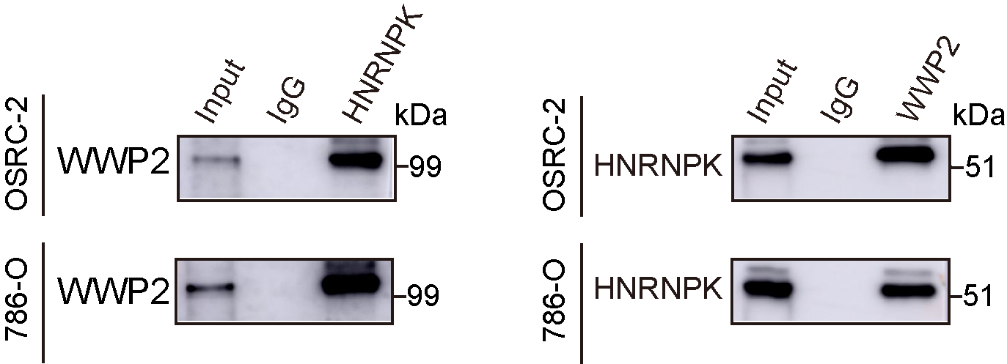


# Figure S10. Co-IP assays showed endogenous interaction between WWP2 and HNRNPK.

**
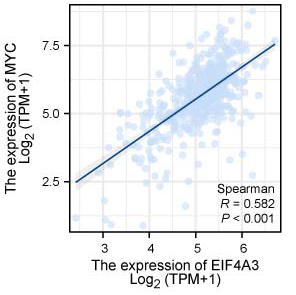
**

# Figure S11. Expression of c-MYC is positively related to EIF4A3 expression.
